# Lipopolysaccharide priming enhances expression of effectors of immune defence while decreasing expression of pro-inflammatory cytokines in mammary epithelia cells from cows

**DOI:** 10.1186/1471-2164-13-17

**Published:** 2012-01-12

**Authors:** Juliane Günther, Wolfram Petzl, Holm Zerbe, Hans-Joachim Schuberth, Dirk Koczan, Leopold Goetze, Hans-Martin Seyfert

**Affiliations:** 1 Leibniz Institute for Farm Animal Biology (FBN), Wilhelm-Stahl-Allee 2, D-18196 Dummerstorf, Germany; 2 Clinic for Ruminants, Ludwig-Maximilians-University Munich, Sonnenstr. 16, D-85764 Oberschleißheim, Germany; 3 Institute of Immunology, University of Veterinary Medicine, Bischofsholer Damm 15, D-30173 Hannover, Germany; 4 Institute for Immunology, Medical Faculty, University of Rostock, Schillingallee 70, D-18055 Rostock, Germany; 5 Pfizer Animal Health, Linkstr. 10, D-10785 Berlin, Germany

## Abstract

**Background:**

Udder infections with environmental pathogens like *Escherichia coli *are a serious problem for the dairy industry. Reduction of incidence and severity of mastitis is desirable and mild priming of the immune system either through vaccination or with low doses of immune stimulants such as lipopolysaccharide LPS was previously found to dampen detrimental effects of a subsequent infection. Monocytes/macrophages are known to develop tolerance towards the endotoxin LPS (endotoxin tolerance, ET) as adaptation strategy to prevent exuberant inflammation.

We have recently observed that infusion of 1 μg of LPS into the quarter of an udder effectively protected for several days against an experimentally elicited mastitis. We have modelled this process in primary cultures of mammary epithelial cells (MEC) from the cow. MEC are by far the most abundant cells in the healthy udder coming into contact with invading pathogens and little is known about their role in establishing ET.

**Results:**

We primed primary MEC cultures for 12 h with LPS (100 ng/ml) and stimulated three cultures either 12 h or 42 h later with 10^7^/ml particles of heat inactivated *E. coli *bacteria for six hours. Priming-related alterations in the global transcriptome of those cells were quantified with Affymetrix microarrays. LPS priming alone caused differential expression of 40 genes and mediated significantly different response to a subsequent *E. coli *challenge of 226 genes. Expression of 38 genes was enhanced while that of 188 was decreased. Higher expressed were anti-microbial factors (β-defensin *LAP, SLPI*), cell and tissue protecting factors (*DAF, MUC1, TGM1, TGM3*) as well as mediators of the sentinel function of MEC (*CCL5, CXCL8*). Dampened was the expression of potentially harmful pro-inflammatory master cytokines (*IL1B, IL6, TNF-α*) and immune effectors (*NOS2*, matrix metalloproteases). Functional network analysis highlighted the reduced expression of *IL1B *and of *IRF7 *as key to this modulation.

**Conclusion:**

LPS-primed MEC are fitter to repel pathogens and better protected against misguided attacks of the immune response. Attenuated is the exuberant expression of factors potentially promoting immunopathological processes. MEC therefore recapitulate many aspects of ET known so far from professional immune cells.

## Background

Inflammation of the mammary gland (mastitis) caused by environmental pathogens is a serious problem in dairy industry. Infection with *Escherichia coli *(*E. coli*) often provokes severe inflammation, greatly reduces milk yield and may eventually cause heavy tissue damage in the mammary gland [1,2]. Various attempts were made in the past to reduce both, incidence and severity of mastitis. These included measures promoting effective pathogen clearance and preventing an exuberant pathological inflammatory reaction. Prophylactic immunization of cows with *E. coli *J5 bacterin as a vaccine consistently reduced the severity of the disease [3,4]. However, it emerged during the last years that such vaccinations do not reduce the rate of new infections [5-7]. Alternatively, the innate immune response of the udder was primed by initiation of an inflammation. Therefore, the udder was infused with non-pathogenic bacteria [8] or the strong immune stimulant lipopolysaccharide (LPS). This reduced the severity of a subsequently elicited mastitis [9] and was found to even protect shortly after endotoxin infusion against an experimentally elicited mastitis [10]. The mechanisms underpinning these potentially beneficial effects are unknown.

A strong primary bacterial insult may cause reduced immune responsiveness of the host to subsequent pathogen challenges. This phenomenon known as endotoxin tolerance (ET) was widely examined *in vivo *in mouse and human during sepsis. Monocytes and macrophages from those species have been used to study relevant mechanisms *in vitro *[11,12] (see [13] for a review). Endotoxin tolerant monocytes exhibit decreased inducibility of pro-inflammatory cytokine synthesis coupled with an upregulated synthesis of anti-inflammatory cytokines as well as increased phagocytosis [14]. These features contribute to protect against septic shock and promote efficient bacterial clearance in the case of a re-infection. On the other hand, ET impairs antigen presentation of these cells through strong and long-lasting reduced expression of several major histocompatibility complex class II molecules (MHC II) [14,15]. Impairment of TLR-signalling by the induction of negative regulators, plasticity in the dimer composition of the transcription factor NF-κB [12] as well as epigenetic chromatin modification at promoters of different immune relevant genes contribute to the phenotype of endotoxin-tolerance in monocytes/macrophages [11]. Little is known about the impact of other cell types for establishing the ET phenotype.

Epithelial cells form the first line of defence against invading pathogens. The mammary epithelial cells (MEC) line the large surface area of the highly branched milk parenchyma of the mammary gland. MEC outnumber by far any other cell type possibly coming into contact with pathogens invading the lactating mammary gland. These cells are not only producing the milk but contribute significantly to the immune defence of the mammary gland [16,17]. They exert sentinel as well as effector functions of immune defence. Pathogen contact or stimulation with LPS endotoxin may elicit the expression of a battery of cytokines/chemokines and also of factors directly fighting off pathogens. These include the bactericidal β-defensins (lingual antimicrobial peptide LAP, bovine neutrophil β-defensin BNBD5) [18,19], some complement factors, and acute phase proteins [16,17,20-22] collectively referred to as "effectors" of innate immune defence.

Our group has recently found that a mild transient stimulation of healthy udders with a single low dose of LPS (1 μg/quarter) will not only reduce the severity of a subsequently elicited *E. coli *mastitis, but will protect the udder from colonization with *E. coli *pathogens for three to ten days [23]. In order to analyze mechanisms of inducible immune-protection in the udder, we used primary bovine MEC (pbMEC) as an established cell model for monitoring udder relevant immune functions [17]. Cultures were primed with low-doses of LPS and changes in their global transcriptome were measured with microarrays. Of particular interest was the evaluation of LPS priming-related alteration of the MEC response towards a subsequent challenge with *E. coli*. We show that LPS priming enhances expression of bactericidal and cell protective factors but attenuates pathogen induced expression of pro-inflammatory cytokines.

## Results

### Profiling the LPS-mediated modulation of the global transcriptome in MEC

**Figure 1:**
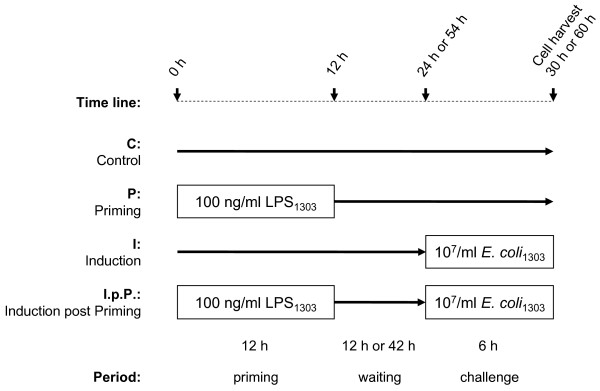
**Schematic diagram of the experimental setting**.

Cultures of primary epithelial cells (pbMEC) were used to globally profile the effects of LPS on the transcriptome. The cells were stimulated (primed) for 12 h with a low concentration of LPS (100 ng/ml) and their RNA was collected after either a short (18 h) or an extended (48 h) waiting period (Figure 1). Respective controls of untreated cultures were included. For monitoring the effect of LPS priming upon the immune responsiveness of those cells, we challenged the cultures for 6 h with 10^7^/ml of *E. coli *particles either 12 h or 42 h after the LPS pre-treatment. Thus, cells were either harvested 30 h (short waiting experiment) or 60 h (long waiting experiment) after start. Adequate control cultures of non-primed cells challenged with the same *E. coli *stimulus were also analysed. The entire experiment therefore consisted of eight different conditions. It was replicated with pbMEC cultures obtained from three different cows. Thus in all 24 RNA samples were collected and individually analysed with GeneChip bovine genome arrays (Affymetrix).

### LPS priming induced changes level off after time

**Figure 2:**
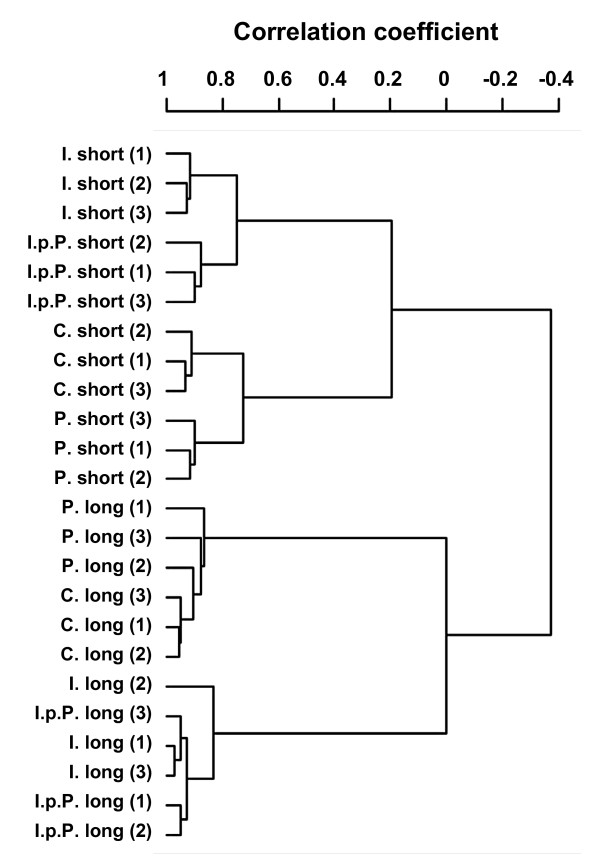
**Dendrogram clustering the data from the individual experimental cultures, using centred correlation and average linkage**. Numbers indicate pbMEC preparations obtained from the three different cows. The dendrogram is based on all data as obtained after the GCRMA normalization. Note that the data are clustering according to treatment rather than the individuals from which the pbMEC cultures have been established.

The correlation analysis of the microarrays (Figure 2) revealed that the three biological replicates clearly clustered together in any of the respective treatment groups if the cells were collected 18 h after the LPS treatment. This was no longer observed if the cells were analysed after extending the waiting period to 48 h post priming. Hence, the LPS effect vanishes with time.

In the short waiting experiments we found that priming modulated the abundance of 48 transcripts (37 higher and 11 lower; Table 1) relative to the control. The expression of 317 transcripts was significantly different between primed and non-primed cells subsequent to an *E. coli *challenge. Priming increased the expression from 45 of these transcripts while decreasing it from 272 of them (table 1; I.p.P. *vs*. I.). It was possible to identify from these 317 differentially expressed transcripts (DET) 226 differentially expressed annotated genes (DEG; Table 1).

**Table 1:** Total numbers of significantly differentially expressed transcripts (DET) and genes (DEG) comparing LPS-primed (P.) *vs*. unstimulated pbMEC (C.) and *E. coli *challenged primed (I.p.P.) *vs. E. coli *challenged naïve cells (I.)

	Short waiting period				Long waiting period			
	**P. *vs*. C**.		**I.p.P. *vs*. I**.		**P. *vs*. C**.		**I.p.P. *vs*. I**.	

DET	**48**		**317**		**15**		**6**	
	↑ 37	↓ 11	↑ 45	↓ 272	↑ 11	↓ 4	↑ 2	↓ 4
IPA mapped DEG	**40**		**226**		**13**		**6**	
	↑ 30	↓ 10	↑ 38	↓ 188	↑ 9	↓ 4	↑ 2	↓ 4

Challenging the cultures 48 h post-LPS-priming modulated the abundance of only 15 DET including 13 DEG compared to the controls. Considering the modulation of the immune responsiveness through LPS priming we found that this treatment regulated only six genes differently, if the *E. coli *challenge was applied 48 h after the end of the LPS priming (Table 1). Therefore, we focused our analysis on data obtained from the short waiting experiments. We identified the human orthologs of those bovine DEGs and only considered the human identifiers for the subsequent analyses, since more comprehensive knowledge is available about the human factors regarding their functions and regulatory relationships.

A complete list of significantly regulated genes and all statistically relevant parameters is given in the Additional material (Additional file 1, Table S1 for the comparisons primed *vs*. control and Additional file 2, Table S2 for induced post priming *vs*. induced).

### Priming of pbMEC induced the expression of genes involved in immune-protective mechanisms

Priming alone enhanced the expression of 30 genes in pbMEC. The functional analysis of these genes with the Ingenuity pathway analysis software (IPA) attributed to them the category "Inflammatory Response" (including 10 factors, p = 9.53 × 10^-7^) with highest statistical significance. Priming most strongly enhanced the abundance of the mRNA encoding the chemokine CCL5 (466-fold, Table 2). This factor recruits monocytes, T cells and eosinophils but is also known to be anti-apoptotic for macrophages. Priming also strongly enhanced the expression of the acute phase response factors serum amyloid A3 (*SAA3*), haptoglobin (*HP*), complement factor B (*CFB*), lactotransferrin (*LTF*), and of the membrane protection factor mucin 1 (*MUC1*). Moreover, priming increased the mRNA abundance of a whole battery of factors known to be involved in antigen processing (leukocyte-derived arginine aminopeptidase, *ERAP2*; cathepsin S, *CTTS*) and presentation (MHC orthologs of *HLA-DRA, HLA-DQA1, HLA-DQB1, HLA-DMB, HLA-A, HLA-DMA*; Table 2 and Additional file 1, Table S1A).

**Table 2:** Top ten DEG after short waiting period comparing LPS-primed (P.) *versus *unstimulated control cells (C.)

Symbol	Entrez Gene Name	Mean ratio	Expression regulated by*			
		**P./C**.	IL1	TNF	IL6	IRF7
**Priming enhances expression of:**						
CCL5	chemokine (C-C motif) ligand 5	**466**	x	x	x	x
SAA3	serum amyloid A3	**146**	x			
FAM14A	family with sequence similarity 14, member A	**21**				
HP	haptoglobin	**14**	x	x	x	
RTP4	receptor (chemosensory) transporter protein 4	**13**				
PARM1	prostate androgen-regulated mucin-like protein 1	**8**				
CFB	complement factor B	**7**		x		
PLAC8	placenta-specific 8	**7**				
HLA-DRA	major histocompatibility complex, class II, DR alpha	**6**	x			
MUC1	mucin 1, cell surface associated	**5**	x	x		
**Priming decreases expression of:**						
ELTD1	EGF, latrophilin and seven transmembrane domain containing 1	**-3.5**				
TRIB3	tribbles homolog 3	**-2.1**				
TBXAS1	thromboxane A synthase 1	**-2.0**				
RASSF4	Ras association domain family 4	**-2.0**				
PYCR1	pyrroline-5-carboxylate reductase 1	**-1.9**				
G0S2	putative lymphocyte G0/G1 switch gene	**-1.9**	x	x		
FUT1	fucosyltransferase 1	**-1.8**				
C1orf24	chromosome 1 open reading frame 24	**-1.8**				
PCTP	phosphatidylcholine transfer protein	**-1.6**				
PSAT1	phosphoserine aminotransferase 1	**-1.6**				

* Expression of the respective gene is known to be regulated by IL1, TNF, IL6, and/or IRF7 (based on Ingenuity Knowledge Database).

Priming decreased the expression of only ten genes. Overall, the magnitude of downregulation was very low. The mRNA concentration of the most downregulated gene *ELTD1 *(EGF, latrophilin and seven transmembrane domain containing 1) was in primed cultures only 2/3^rd ^of that found in the control cells (Table 2). This gene encodes a G-protein coupled receptor with unknown function. Three other downregulated genes are known to increase "Apoptosis of Cell Lines" (tribbles homolog 3, Ras association domain family 4, and putative lymphocyte G0/G1 switch gene 2; Table 2).

Only nine genes were found to be differentially expressed relative to the control if the waiting period after the LPS stimulus was extended to 48 h. Priming enhanced their expression. Three of those genes were also found to be regulated after the short waiting period. The latter genes encode CCL5, LTF, and transglutaminase 3 (TGM3). The extent of priming-induced upregulation of *CCL5 *and *LTF *expression was weaker in the long waiting experiment (36- and 2-fold, respectively; Additional file 1, Table S1B). However, it was stronger for *TGM3 *(7-fold after long- and 2-fold after short waiting period; Additional file 1, Table S1B). The mRNAs encoding interleukin IL-6 and the chemokine CXCL6 were upregulated by priming only after the long waiting period (6- and 4-fold; Additional file 1, Table S1B).

### Priming enhanced the *E. coli *mediated induction of immune-protective molecules, but decreased expression of pro-inflammatory and cell death associated factors

We have previously reported that an *E. coli *challenge of naïve pbMEC regulated the expression of > 300 genes [22]. Given this background knowledge, we analyse in this study only the LPS priming-mediated modulation of the *E. coli*-elicited response.

**Figure 3:**
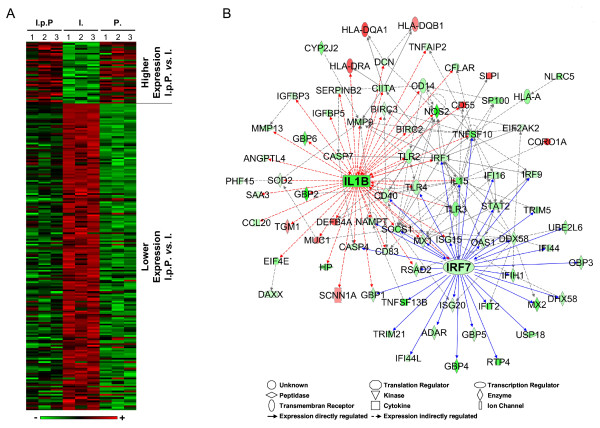
**Overview of differentially expressed genes in primed and challenged pbMEC (I.p.P.) compared to naïve cells challenged with the same *E. coli *stimuli (I.)**. **A: **Hierarchical clustering of the 226 DEGs comparing primed and challenged cells to naïve challenged pbMEC. The heat map represents the expression level of each DEG in the three different pbMEC preparations (1, 2, 3) as determined in the three challenge groups (I.p.P., I., and P.). Data were sorted according to the extent of the differential expression between I.p.P. *vs*. I. The heatmap is based on the log(2) fold changes between the 3 treatment groups relative to the respective un-stimulated control cells (Additional file 2, Table S2A). The rows of the matrix were normalized to have the values 0 as a mean, and 1 as associated variance. Red indicates high and green low expression. **B: **IPA network analysis of the regulatory relationship of DEGs between the treatment groups I.p.P. (induction post priming) *vs *I. (challenge of naïve cells). Red, higher expression in I.p.P.; green, lower expression in I.p.P. IL-1B is known to regulate the expression of 44 genes (red arrows). The transcription factor IRF7 is known to regulate the expression of 34 genes (blue arrows).

We observed for 226 genes a priming related modulation of their expression subsequent to an *E. coli *challenge (Table 1; columns I.p.P. *vs*. I.). Expression of 38 genes was higher while that of 188 genes was lower than in non-primed but challenged cells (Figure 3A). We first quantitatively differentiate the impact of the LPS priming upon their subsequent regulation by the *E. coli *challenge. The biological functions of the factors encoded by the DEG were determined using the Ingenuity software. In a second step we identified the molecular relationships of all 226 DEG by an IPA network analysis.

The LPS priming mediated quantitative modulation of mRNA levels eventually affects all different regulatory levels controlling mRNA abundance. Accordingly, we sort the genes into six different regulatory groups.

#### Priming enhanced expression after *E. coli *challenge

We first consider those 38 genes revealing increased mRNA abundance after an *E. coli *challenge compared to non-primed cells.

##### Group1: Priming enhanced induction after an *E. coli *challenge

Sixteen of these 38 genes revealed higher mRNA concentrations than observed in unstimulated control cells (Additional file 2, Table S2A, highlighted in red). Most of them (11 genes) were already induced by priming only (Figure 3A; Table 3 compare columns I.p.P./C. *vs*. P./C.; Additional file 2, Table S2A). Examples include *CORO1A *(coronin, actin binding protein, 1A), *CD55 *(decay accelerating factor for complement; also known as DAF), *SLPI *(secretory leukocyte peptidase inhibitor), and all three MHC II-encoding factors (orthologs of the human factors HLA-DRA, HLA-DQA1, HLA-DQB1). The *E. coli *stimulus did not increase their mRNA abundance any further. However, it did increase the mRNA concentration of the bactericidal β-defensins (*DEFB4*, human ortholog of the bovine β-defensins, notably the lingual antimicrobial peptide [*LAP*]) and those membrane protection factors *TGM3 *(transglutaminase 3) and *MUC1 *(mucin 1) above their already priming-induced increased levels. Interestingly, mRNA levels of two genes with an enhanced expression in primed and *E. coli *challenged cells (*CORO1A, CD55*) were decreased by an *E. coli *challenge of non-primed cells.

**Table 3:** Priming-related modulation of gene expression subsequent to an *E. coli *challenge after a short waiting period

Symbol	Entrez Gene Name	Mean ratio	Mean ratio compared to un-stimulated control (C.)			Expression regulated by****			
		**I.p.P/I**.	I.p.P./C.	I./C.	P./C.	IL1	TNF	IL6	IRF7
**Priming provokes a higher the expression level after an *E. coli *challenge of:**									
CORO1A*	coronin, actin binding protein, 1A	2.8**	2***	*-2*	*2*				
**CD55**	CD55 molecule, decay accelerating factor for complement	**2.6**	*2*	*-2*	*2*	x	x		
**HLA-DQA1**	major histocompatibility complex, class II, DQ alpha 1	**2.3**	*4*	*2*	*4*			x	
TGM3	transglutaminase 3	**2.3**	**4**	*2*	**2**				
**SLPI**	secretory leukocyte peptidase inhibitor	**2.2**	*5*	*2*	*4*		x		
LY6G6E	lymphocyte antigen 6 complex, locus G6E	**2.2**	*2*	NC	*2*				
CYP26A1	cytochrome P450, family 26, subfamily A, polypeptide 1	**2.1**	NC	*-2*	*2*				
**DEFB4A**	defensin, beta 4A	**2.1**	*47*	*22*	*24*	x	x		
**HLA-DRA**	major histocompatibility complex, class II, DR alpha	**2.1**	*6*	*3*	**6**	x			
ATP6V0A4	ATPase, H+ transporting, lysosomal V0 subunit a4	**2.0**	NC	*-2*	NC				
**Priming provokes a lower expression level after an *E. coli *challenge of:**									
**NOS2**	nitric oxide synthase 2, inducible	**-10.7**	*14*	**154**	NC	x	x	x	
**GBP2**	guanylate binding protein 2, interferon-inducible	**-8.1**	**36**	**287**	*10*	x	x	x	
APOL3	apolipoprotein L, 3	**-7.9**	*4*	**30**	NC				
**IL1B**	interleukin 1, beta	**-6.4**	*5*	**32**	NC	x	x		
TIFA	TRAF-interacting protein with forkhead-associated domain	**-6.0**	*6*	**36**	NC				
**TNFSF13B**	tumor necrosis factor (ligand) superfamily, member 13b	**-5.8**	*15*	**89**	NC		x		x
TMEM140	transmembrane protein 140	**-5.8**	*2*	**11**	NC				
**GBP4**	guanylate binding protein 4	**-5.8**	NC	**6**	NC		x		x
LMO2	LIM domain only 2 (rhombotin-like 1)	**-5.3**	NC	**5**	NC				
**RTP4**	receptor (chemosensory) transporter protein 4	**-4.7**	*15*	**72**	*4*				x

* bold letters, part of a network connected by the IPA relationship "Expression" (Fig 3B)

** bold numbers, fc > 1.5, p < 0.005

*** italic number, fc > 1.5, p > 0.005

NC, No change fc < 1.5

**** Expression of the respective gene is known to be regulated by IL1, TNF, IL6, and/or IRF7 (based on Ingenuity Knowledge Database).

##### Group 2: Priming prevented pathogen mediated downregulation

Eighteen genes revealed significantly higher mRNA concentrations in primed and *E. coli *challenged (I.p.P.) than in non-primed challenged cells (I.) although their mRNA concentrations were not changed compared to the unstimulated controls (Table 3; Additional file 2, Table S2A, highlighted in grey). This indicates that priming prevented their downregulation in response to an *E. coli *challenge. The identification of their associated biological functions by IPA analysis revealed that three of those factors are associated with "Molecular Transport" (solute carrier family 34, member 2, ATPase, H+ transporting, lysosomal V0 subunit a isoform 4, and sodium channel, nonvoltage-gated 1 alpha; *p *= 6.29 × 10^-3^) and two factors are involved in "Pyrimidine" as well as "Purine Metabolism" (*CRCP*, Calcitonin gene-related peptide receptor component and *POLRMT*, polymerase [RNA] mitochondrial [DNA directed]).

##### Group 3: Priming reduced pathogen mediated downregulation

The mRNA abundances of four genes were higher in primed and challenged cells compared to non-primed challenged cells because their *E. coli *induced downregulation was diminished by priming (Additional file 2, Table S2A, highlighted in green). These encode the transcription factor CP2-like 1, the heat shock protein HSPB6, the ribosomal modification protein RIMKLB, and the sclerostin domain containing 1. Down regulation of the latter factor in breast ductal epithelial cells is associated with breast cancer [24].

#### Priming provoked a significant lower induction after the *E. coli *challenge

Overall, we found 188 genes with a significantly lower mRNA abundance in response to the *E. coli *stimulus after the short waiting period post-priming.

##### Group 4: Priming prevented pathogen mediated induction

The expression of 30% of the 188 genes with lower mRNA concentration in *E. coli *challenged primed cells compared to non-primed challenged cells (58 genes; Figure 3A; Additional file 2, Table S2A, highlighted in grey) was unchanged compared to the control indicating that priming completely prevented their induction. Fifteen of those genes are associated with the functional category "Inflammatory Disease" (p = 3.71 × 10^-4^) and nine with "Cell Death" (p = 5.7 × 10^-4^). Prominent members of these categories are interleukin *IL15*, toll-like receptor 3 (*TLR3*), the bovine ortholog of the MHC I factor *HLA-L*, the death-domain associated protein *DAXX*, and the MHC II transactivator *CIITA*.

##### Group 5: Priming reduced the pathogen mediated induction

The induction of 106 genes was reduced but not abolished by priming (Additional file 2, Table S2A, highlighted in red). Twenty eight of those genes are associated with the IPA category "Cell Death" forming the category with the highest statistical significance (p = 4.56 × 10^-8^). This category comprises molecules acting downstream of the death receptors (*e.g*. the caspases *CASP4 *and *CASP7*, the CASP8, FADD-like apoptosis regulator *CFLAR*, and the baculoviral IAP repeat-containing factors *BIRC2 *and *BIRC3*). The inducible nitric oxide synthase *NOS2 *belongs to this group, representing a candidate gene for bactericidal functions, but also the similarly regulated genes encoding the pro-inflammatory cytokine IL-1B and encoding the cytokines of the TNF-ligand family (TNFSF13B and TNFSF10). LPS priming-mediated attenuation of their pathogen-mediated induction suggests a general dampening of the inflammatory response. Augmenting this we note that the level of the mRNA encoding the TRAF interacting protein with fork-head-associated domain (TIFA) is also greatly reduced. This factor is an adaptor to the TNF receptor associated factor 6 (TRAF6) as well as to the IL-1 receptor-associated kinase 1 (IRAK1), both known as crucial mediators of TLR-receptor driven NF-κB activation [25]. Thus, LPS priming dampens the pathogen-mediated activation of this signalling cascade known as a key regulator of inflammation.

##### Group 6: Priming enhanced pathogen mediated downregulation

Priming prior to the *E. coli *stimulation enforced the downregulation of 24 genes (Additional file 2, Table S2A, highlighted in green). Seven of them are associated with the IPA category "Cell Death" (p = 1.82 × 10^-3^), including epidermal arachidonate lipoxygenase *ALOX12*, the insulin-like growth factor binding protein *IGFBP5*, and the pseudokinase *TRIB3*. The latter factor may promote cell death but is also known to inhibit lipid metabolism and insulin-mediated activation of the protein kinase AKT.

#### *E. coli *challenge after long time priming

Challenging the cells 42 h after the LPS priming with *E. coli *greatly reduced the LPS effect upon the pathogen response. We found only six genes significantly differentially expressed between the induction post priming and the induction without priming (Table 1; Additional file 2, Table S2B). Two genes revealed a higher and four a lower expression. Their relevance for immune functions has not been reported.

### Identification of central factors regulating the different response to *E. coli *after priming

The expression related interdependence of those 226 priming mediated DEGs was analysed with the IPA software in order to uncover regulatory key factors. A network emerged comprising 78 of those DEGs. It is associated with the IPA functions "Inflammatory Response", "Antimicrobial Response", and "Inflammatory Disease" (Figure 3B). The central node molecules in this network are the cytokine IL-1B and the transcription factor interferon regulatory factor 7 (IRF7). IL-1B is known as a central regulator of immune response and the IPA data base indicates that it may affect the expression of 44 from among our 226 DEGs, including IRF7. This factor is activated through the TRIF-factor dependent downstream signalling of TLR4 and of the endosomal TLR receptors (TLR3, 7, 8, 9) [26]. IRF7 is known to directly influence the expression of 34 DEGs. Other factors occupying node positions in this network are known as DEAD (Asp-Glu-Ala-Asp) box polypeptide 58 (DDX58; linked to 14 downstream factors), IRF1 (linked to 13 downstream factors), and TNFSF10 (linked to 12 downstream factors). The expression levels of all these factors were reduced by the LPS priming prior to the pathogen challenge.

### Validation of the induction post-priming experiment by RT-qPCR

**Figure 4:**
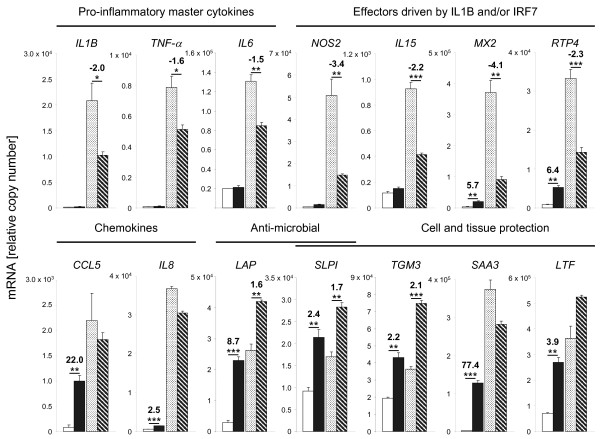
**Quantification of the expression of selected genes by RT-qPCR**. Ordinate, relative copy number (n, 3 each; ± S.E.M.) determined in the short time waiting experiment. Open bars: unstimulated control, filled bars: primed cells, light sanded bars: *E. coli *induction of naïve cells; dark shaded bars: *E. coli *induction of primed cells. Fold changes and significance (* p < 0.1, ** p < 0.05, *** p < 0.01, paired t-test) of the priming-related differential expression are indicated. All individual data are listed in the Additional file 4, Table S4.

Our microarray analysis had shown that priming prior to a pathogen challenge enhances the expression of bactericidal and immune-protective factors while attenuating cytokine and chemokine expression. We used RT-qPCR to validate from the same RNA samples as used for the microarray analysis the differential expression of representative candidate genes (Figure 4; Additional file 3, Table S3 and Additional file 4, Table S4). We included some other key immune regulatory factors (*TNF-α, IL6, CXCL8 *[also known as *IL8*]) factors revealing a statistically insignificant modulation in the microarray experiments under the stringent conditions applied to that analysis. We found a good correlation (r, 0.91; 14 genes) between changes measured by RT-qPCR and the microarray data (Additional file 5, Table S5). The RT-qPCR data show that priming quenched the pathogen induced expression of the cytokines *IL1B, TNF-α*, and *IL6 *by ~30-50%. The mRNA concentrations of these factors are known to eventually peak in MEC within 1 h after an *E. coli *stimulation and are subsequently massively downregulated to lower levels which are sustained from 6 h to 24 h post stimulation [22,27]. Thus, the only moderate modulation after 6 h of pathogen stimulation as observed here is conceivably a conservative estimate of the actual priming-related changes.

IL-1B, TNF-α, and IL-6 are often called "master" cytokines, owing to their secretion and perception by many different cell types and their key role in orchestrating the synthesis of other cytokines and chemokines. TNF-α may eventually influence the expression of 60 of those 226 priming-mediated DEGs while IL-6 might affect the expression of another 27 DEGs (Table 3; Additional file 1, Table S1 and Additional file 2, Table S2). Similarly reduced was the IL-1/IRF7 regulated induction of the factors *NOS2 *(bactericidal and immune regulatory), *IL15, MX2*, and *RTP4*. On the other hand, the RT-qPCR measurements very clearly confirmed that priming alone significantly enhances the expression of the key chemokines (*CCL5, IL8*), of anti-microbial factors (*LAP, SLPI*) and also of molecules which are known to promote cell and tissue protection in the course of an inflammation (*SLPI, TGM3, SAA3, LTF*). The priming-mediated enhanced expression of *LAP, SLPI*, and *TGM3 *after pathogen stimulation was also confirmed.

## Discussion

Priming of udders with LPS protects for a limited time against manifestation of experimentally induced mastitis in mid-lactating cows [10,23]. Mild endotoxin stimulation might thus possibly be developed as a means to reduce both, incidence and severity of mastitis in critical periods of the lactation cycle. We have undertaken the current study to learn more about the broader implications of a mild endotoxin pre-stimulation (priming) of primary MEC cultures upon their subsequent reaction towards a challenge with heat inactivated *E. coli *particles. We have previously reported on relevance and some limitations of the MEC model to study mastitis related immune regulations, on the *E. coli *challenge as also applied here and on the general techniques of our microarray analysis [17,18,22,27,28]. We based the current study on using primary MEC cultures from three different cows. Using this small number of biological replica might limit the statistical power. However, we have previously documented that the intra-animal variability of pathogen induced gene expression between those cultures is much less than observed between cows [22]. We discuss our key observations in terms of priming (i) enforced sentinel functions of the MEC, (ii) improved protection against pathogens and tissue damage and (iii) reduced expression of master cytokines and potentially harmful factors limiting the risk of induced immune-pathology.

### Priming enforces sentinel functions of the MEC

A mild LPS priming (1 μg/udder quarter, for 12 h) was found to significantly increase *in vivo *the number of somatic cells in udders [23]. Thus, it was not surprising to find in our *in vitro *pbMEC model that LPS priming enhanced quite strongly the level of mRNAs encoding the chemokines CCL5 (also known as RANTES) and CXCL8. Both factors are known to trigger diapedesis of mononuclear cells, T-cells and macrophages (CCL5 [29]) and polymorph nuclear granulocytes (PMN; CXCL8 [30]) through the endothelia of the blood vessels recruiting them into the inflamed sites. Elevated levels of both factors enhance the abundance of professional sentinel cells (*e.g*. macrophages) in the udder and conceivably improve the competence of the gland to counter act recurrent infections. It is known from macrophages that LPS priming enhances CCL5 expression in response to a second LPS stimulus [11].

In contrast to observations made on endotoxin tolerant macrophages [14,15], we found that priming increased the abundances of a variety of mRNA moieties encoding peptidases for antigen processing (ERAP2, CTTS) and MHC-II factors (bovine orthologs of HLA-DQA1, -DRA, -DQB1) in MEC. While MEC are generally not considered as professional antigen-presenting cells (APCs) it is known that alveolar epithelial cells express MHC-II factors and are relevant APCs in the lung [31]. Thus, enhanced expression of these factors in LPS-primed MEC suggests an improved readiness of the primed cells for antigen presentation and thus improving their sentinel competence.

### Priming improves protection against pathogens and tissue damage

Priming increased the levels of mRNAs encoding the bactericidal β-defensin lingual antimicrobial peptide (LAP). It was shown in macrophages that ET remodels the chromatin at the promoters of such "not LPS tolerant" effector genes of immune defence through differential histone modification making them more quickly responsive to a second stimulus [11]. Epigenetic mechanism might also operate during ET at the *LAP *promoter of the MEC, since mastitis induced expression of the *LAP *gene requires chromatin decompaction at the promoter [32].

Focussing on LAP as an example for a β-defensin encoding gene serves only as a paradigm for the regulation of this class of molecules, which are so abundantly encoded in the bovine genome. More than 100 highly related copies of β-defensin-encoding genes were uncovered in the analysis of the entire bovine genome [33]. The infection induced expression of *LAP *and other β-defensins in the udder has previously been reported [18,19,34]. The wealth of these β-defensin-encoding genes could not be analysed with the tools applied in this study, but LPS priming-enhanced abundance of the LAP-encoding RNA might be indicative for the regulation of other members of this gene family. Hence, LPS priming supports protection against colonization of the udder by bacterial pathogens. This was indeed found to be the case *in vivo *[10,23].

Priming increased also the mRNA level of the defensin like factor secretory leukocyte peptidase inhibitor (SLPI), known as contributing to the defence against bacteria, fungi and retroviruses in epithelial tissues [35]. This multifunctional serine protease inhibitor protects epithelial tissue during inflammation from the attack by endogenous proteolytic enzymes. Moreover, SLPI is known to bind and synergize with proepithelin. This growth factor promotes proliferation of epithelial cells and suppresses activation of neutrophils. SLPI abrogates proteolytic cleavage of proepithelin into the inflammation sustaining factor epithelin [36]. Lactotransferrin (LTF) is another multifunctional factor revealing LPS priming-related upregulated mRNA levels. This factor has bacteriostatic, bactericidal and some immune modulating properties, but also the capability to bind LPS (reviewed in [37]). It was shown that LTF pre-treatment protects mice from pathophysiological effects of LPS and enhances the survival after an LPS injection [38]. Priming enhanced the levels of the mRNA encoding the complement factor CFB. This protein activates the alternative complement pathway thereby strengthening unspecific immune defence mechanisms. Expression of this factor in the udder is also strongly induced during *E. coli*-elicited mastitis [17].

Priming increased the mRNA abundances of several factors protecting against membrane damage associated with the so called "bystander killing" effect of activated immune defence. These include the decay accelerating factor for complement CD55 (also known as DAF) protecting membranes of the host cells against misguided attacks through complement factors and also the glycoprotein mucin 1 (MUC1). This mucin is a key component of the apical (luminal) membrane of the MEC [39] forming a physical barrier against microbial attacks [40]. Increased expression of the transglutaminases *TGM1 *and *TGM3 *indicates stabilization of tissues and cells by protein cross-linking [41]. These factors contribute also to the formation of tight junctions between mammary epithelial cells and are known to promote cross linking of proteins in the extracellular matrix thereby initiating wound healing.

### Priming reduces expression of master cytokines and of potentially harmful factors

It is known from macrophages that endotoxin tolerance (ET) down-regulates the expression of the inflammatory master cytokines tumor necrosis factor α (TNF-α), interleukin 1 (IL-1) and IL-6 (reviewed in [13]). They all belong to the class of "LPS-tolerant" genes which become refractory in macrophages to repeated LPS stimulation [11]. We now made a similar observation in the MEC. LPS priming moderately, yet significantly reduces their mRNA levels after a subsequent *E. coli *challenge in these cells. These three factors together orchestrate and dominate many aspects of the local as well as systemic immune response [42,43]. While their adequate induction through invading pathogens is indispensable for mounting a productive immune defence in the host, their overshooting expression may be detrimental. Confining their expression is therefore an overarching beneficial effect of ET and contributes to prevent immune-pathology. Our data suggest that this principle applies also to the regulation of the immune defence in the udder mediated through MEC. Moreover, we validated reduced and confined expression for some of their secondary response factors during the subsequent pathogen challenge. These factors include the nitric oxide synthase *NOS2*. This enzyme produces nitric oxide radicals, which are bactericidal but also harmful to the host cells and tissues as well. Similarly, expression of the metallopeptidases (*MMP9, MMP13*) was found to be confined by LPS priming. These proteases may disintegrate the extra-cellular matrix and connectives. LPS priming appears to prohibit their exuberant production and thus reduces activity of these potentially harmful factors.

Expression of many factors associated with cell death was found to be limited by LPS priming. These factors include the caspases *CASP4 *and -*7, DAXX, CFLAR *to name only some of them. Reduced and limited induction of these factors is conceivably a consequence of reduced TNF-α induction and indicates improved preservation of cell- and tissue-integrity.

Reduced and dampened induction of the TLR/NF-κB axis of pathogen signal transduction appears to be crucial for the ET mediated modulation of the immune response in the MEC. Not only was the pathogen stimulated expression of several TLRs reduced (*TLR2*, -*3*,-*4*) but also were factors induced by the LPS priming which are known to interfere with NF-κB activation, including the coronin *CORO1A *and *SPLI*. CORO1A is known to suppress TLR2, TLR3, TLR4 and TNF-α mediated NF-κB activation as well as IFN-β promoter activation [44]. Indeed, we found that the mRNA expression of various type I interferon response factors was decreased by priming (*e.g*. myxovirus resistance 2 [*MX2*], receptor transporter protein 4 [*RTP4*], 2'-5'-oligoadenylate synthetase 1 [*OAS1*], interferon stimulated exonuclease gene 20 kDa [*ISG20*]*)*. The factor SLPI has not only a broad spectrum of antimicrobial activities but also anti-inflammatory/immunomodulatory functions [45]. SLPI interferes also with TLR-4 mediated NF-κB activation by inhibiting the interaction between CD14 and LPS [46] thus quenching the production of inflammatory factors like TNF-α, NOS2, COX2, and MMPs [47,48].

## Conclusions

Our global transcriptome assay shows that the MEC recapitulates many aspects of LPS-induced ET which were known so far only from macrophages/monocytes. These features eventually protect the cow from detrimental effects of an overshooting immune response elicited in that very large organ, the udder. Moreover, analysis of our model cells revealed that LPS priming enforces the production of anti-microbial factors, protects integrity of the epithelial cells through enforced tissue stabilization and wound healing. Our data collectively reveal mechanisms underpinning the observation made *in vivo *that LPS pre-stimulation of the udder protects against *E. coli *elicited mastitis.

## Methods

### Cell culture

Primary cultures of MEC (pbMEC) were obtained from udders of three healthy, pregnant (day 130 of gestation) cows in the mid of their first lactation. Cell preparation and their culture on collagen coated dishes were as previously described [17]. The pbMEC growth medium (GM) was RPMI 1640 (Biochrom AG; Berlin, Germany) supplemented with prolactin, hydrocortisone, insulin and 10% FCS as described [18]. Fibroblasts were selectively removed by repeated trypsinization. We number throughout the cultures derived from those three individual cows as 1, 2 or 3.

### *E. coli *1303 *and LPS preparation*

Heat killed (60°C, 30 min) particles of the mastitis causing *E. coli *strain 1303 [19] have been prepared as described [17]. LPS was prepared from this strain by butanol extraction and hydrophobic interaction chromatography [49] followed by a purification with triethylamine and deoxycholate [50] (kindly provided by Sonja von Aulock, University of Konstanz, Germany). It was dissolved in GM (1 mg/ml) by ultra-sonic dispersion (2 min) and aliquots were stored at -20°C.

### LPS priming and challenge with heat inactivated *E. coli *particles

Figure 1 illustrates the experimental settings. Each experiment was performed in triplicate using separate pbMEC cultures (80% confluence) prepared from the udders of those three different cows. Controls (C.) were cultured for 12 h in GM, washed 3× with PBS and cultivated for additional 18 h (short waiting experiment) or 48 h (long waiting experiment). For the priming experiment (P.) the pbMEC cultures were stimulated ("primed") at time 0 h with 100 ng/ml LPS, washed three times with PBS and cultivated for another 18 h or 48 h. For the induction trial (I.) cells were challenged with 10^7^/ml particles of heat killed *E. coli*_1303 _for 6 h. The cultures were handled exactly like the control group (C.) prior to the *E. coli *challenge. For the induction post priming experiment (I.p.P.) cultures were primed for 12 h with LPS, just as described for the priming group (P.). After 3× washing with PBS and waiting for 12 h or 42 h in GM the I.p.P. cultures were also challenged with 10^7^/ml particles of *E. coli*_1303_. Cells were harvested at time 30 h (short waiting experiments) or 60 h (long waiting experiments) and total RNA was prepared.

### Microarray hybridisation

Total RNA for the microarray analysis was extracted with the RNeasy kit exactly as described by the manufacturer (Qiagen, Hilden, Germany). RNA processing, labelling and hybridization used the respective reagent kits from Affymetrix and was exactly done as previously described [22]. Briefly, 5 μg total RNA of each sample was used for cRNA preparation and labelling with the Affymetrix GeneChip^® ^Expression 3' Amplification One-Cycle Target Labelling Kit (Affymetrix, St. Clara, USA). The fragmented cRNA was hybridized for 16 hours at 45°C to GeneChip^® ^Bovine Genome (Affymetrix). The Microarrays were scanned at 1.56 micron resolution using the GeneChip Scanner 3000 7G (Affymetrix). The microarray data sets were submitted to the Gene Expression Omnibus (GEO) database (accession no. GSE32186).

### Microarray analysis

The microarray data were analysed using the Biometric Research Branch (BRB) array tools version 4.1 [http://linus.nci.nih.gov/BRB-ArrayTools.html]. Background correction and normalization of the expression values was performed using the GC Robust Multi-array Average (GCRMA) algorithm [51]. Transcripts were defined as significant differentially expressed (DET) among Control (C.) and Priming (P.) as well as among Induction (I.) and Induction post Priming (I.p.P.) groups if their fold change exceeded > 1.5 and the p-value of the univariate t-test between values paired according the pbMEC preparation was < 0.005. False discovery rates (FDR) values were calculated and are listed in the additional files (Additional file 1, Table S1 and Additional file 2, Table S2). However, this parameter was not applied as a cut off criterion since it would have been too stringent for some comparisons, resulting in no significantly regulated genes at all. This applies in particular to the comparison between primed *vs*. control cultures. Human orthologs of differentially expressed genes (DEG) were determined as described previously [22,52]. Gene ontology and network analysis was performed using the Ingenuity Pathways Analysis (IPA 9.0) software (Ingenuity Systems, Redwood City, CA). This program identifies the biological functions that were most significant to a data set using Fisher's exact test. The calculated p-value specifies the probability that each biological function assigned to a gene list is solely due to chance. The heatmap was generated using the software tool matrix2png [53].

### Quantitative real-time PCR (RT-qPCR)

Total RNA extraction with Trizol (Invitrogen, Karlsruhe, Germany), cDNA preparation with Superscript II (Invitrogen) and quantification of mRNA copy numbers with the LightCycler instrument and the SYBR Green plus reagent kit (both from Roche, Basel, Switzerland) was as described [54]. Quality of the PCR products obtained in those LightCycler runs was validated by subcloning the amplicons into pGEM-Teasy (Promega) and sequencing. Relative copy numbers were titrated against external standards consisting of a dilution series (10^6 ^to 10 copies) of those sequenced plasmids. Sequences of the oligonucleotide primers used are given in Additional file 6, Table S6. Significance of differences was assessed with the t-test of paired values. Spearman's Rank Correlation was used to compare microarray and RT-qPCR measurements using the SigmaStat 3.5 software (Systat Software Inc., San Jose, California, USA).

## Authors' contributions

JG contributed to designing the experiments, analyzed and interpreted the data, and drafted the manuscript. WP contributed to the experimental design, data analysis, and drafting the manuscript. HZ, HJS and LG contributed to conception of the study and critical data interpretation. DK prepared the microarray hybridizations. HMS designed and supervised the study and helped drafting the manuscript. All authors read and approved the final manuscript.

## Supplementary Material

Additional file 1 **Table S1: All DEG from comparison Priming (P.) *versus *Control (C.)**. A) Short time waiting experiment (40 IPA mapped DEG). B) Long time waiting experiment (13 IPA mapped DEG) Click here for file

Additional file 2 **Table S2: All DEG from comparison Induction post Priming (I.p.P.) *versus *Induction (I.)**. A) Short time waiting experiment (226 IPA mapped DEG). B) Long time waiting experiment (6 IPA mapped DEG) Click here for file

Additional file 3 Table S3: Comparison of RT-qPCR and microarray measurements of selected candidate genes Click here for file

Additional file 4 **Table S4: All RT-qPCR values (relative mRNA copy numbers) contributing to Figure 4**. Click here for file

Additional file 5 Table S5: Correlation of relative mRNA concentrations determined in microarray hybridizations or RT-qPCR from three biological pbMEC replica of the four challenge groups (C., P., I., and I.p.P.) of the short and long waiting experiments Click here for file

Additional file 6 Table S6: Sequences of oligonucleotide primers used for real-time PCR quantification Click here for file
