# Supplementary material for: Lipopolysaccharide priming enhances expression of effectors of immune defence while decreasing expression of pro-inflammatory cytokines in mammary epithelia cells from cows
Source: BMC Genomics. 2012 Jan 12;13:17. doi: 10.1186/1471-2164-13-17 (PMC3315725; doi:10.1186/1471-2164-13-17)
Supplement: Additional file 1 — Table S1: All DEG from comparison Priming (P.) versus Control (C.). A) Short time waiting experiment (40 IPA mapped DEG). B) Long time waiting experiment (13 IPA mapped DEG) [file 1471-2164-13-17-S1.PDF]

Table S1: All DEG from comparison Priming (P.) *versus* Control (C.)

A) Short time waiting experiment (40 IPA mapped DEG)

Probe set	Gene symbol	Description	Mean fold change (P./C.)	Parametric *p*-value	FDR	Expression regulated by (IPA)*				fold change of preparation:		
						**IL1** (9)	**TNF** (9)	**IL6** (4)	**IRF7** (3)	**1**	**2**	**3**
**Priming enhanced expression of:**												
Bt.552.1.S1_at	CCL5	chemokine (C-C motif) ligand 5	**466.48**	0.00028	0.13800	x	x	x	x	572.45	414.15	428.16
Bt.278.1.S1_at	SAA3	serum amyloid A3	**146.09**	0.00087	0.16500	x				188.45	113.38	145.92
Bt.11259.1.S1_at	IFI27L2	interferon, alpha-inducible protein 27-like 2	**21.12**	0.00310	0.18400					27.59	15.40	22.16
Bt.12553.1.S1_at	HP	haptoglobin	**14.25**	0.00211	0.18300	x	x	x		14.55	11.42	17.42
Bt.24813.1.A1_at	RTP4	receptor (chemosensory) transporter protein 4	**13.33**	0.00276	0.18300					14.76	10.17	15.77
Bt.24157.1.A1_at	PARM1	prostate androgen-regulated mucin-like protein 1	**7.51**	0.00102	0.16500					7.78	6.63	8.23
Bt.13542.1.S1_at	CFB	complement factor B	**6.85**	0.00058	0.16500		x			6.27	7.01	7.33
Bt.15912.1.S1_at	PLAC8	placenta-specific 8	**6.78**	0.00232	0.18300					5.65	7.32	7.56
Bt.8552.1.S1_at	HLA-DRA	major histocompatibility complex, class II, DR alpha	**6.38**	0.00293	0.18300	x				5.40	6.31	7.64
Bt.9561.1.S1_at	MUC1	mucin 1, cell surface associated	**5.41**	0.00089	0.16500	x	x			5.65	4.89	5.72
Bt.4802.1.S1_at	LTF	lactotransferrin	**5.13**	0.00018	0.11300					5.15	4.93	5.32
Bt.8906.1.S1_at	LGALS9	lectin, galactoside-binding, soluble, 9	**4.54**	0.00369	0.19400					4.59	3.85	5.29
Bt.4751.2.S1_a_at	HLA-DQA1	major histocompatibility complex, class II, DQ alpha 1	**4.16**	0.00336	0.18600			x		3.92	4.90	3.75
Bt.13628.2.S1_a_at	TGM3	transglutaminase 3	**3.09**	0.00013	0.11100					3.06	3.05	3.17
Bt.16118.1.S1_at	CLEC12A	C-type lectin domain family 12, member A	**2.69**	0.00280	0.18300					2.96	2.47	2.66
Bt.22056.2.S1_a_at	ABHD1	abhydrolase domain containing 1	**2.43**	0.00098	0.16500					2.30	2.47	2.52
Bt.350.1.S1_x_at	HLA-DQB1	major histocompatibility complex, class II, DQ beta 1	**2.38**	0.00055	0.16500					2.29	2.45	2.40
Bt.28068.1.S1_at	SLC5A1	solute carrier family 5 (sodium/glucose cotransporter), member 1	**2.37**	0.00114	0.16800					2.51	2.28	2.33
Bt.3762.1.A1_at	HEATR1	HEAT repeat containing 1	**2.28**	0.00258	0.18300					2.47	2.21	2.16
Bt.9360.1.S1_at	S100A8	S100 calcium binding protein A8	**2.02**	0.00407	0.19500	x	x		x	1.97	2.20	1.89
Bt.1007.1.S1_at	HLA-DMB	major histocompatibility complex, class II, DM beta	**1.98**	0.00157	0.18300					1.88	2.01	2.06
Bt.25649.1.A1_at	ERAP2	leukocyte-derived arginine aminopeptidase	**1.77**	0.00090	0.16500					1.79	1.82	1.71
Bt.27760.1.S1_at	HLA-A	major histocompatibility complex, class I, A	**1.75**	0.00185	0.18300					1.78	1.67	1.81
Bt.7938.1.S1_at	CTSS	cathepsin S	**1.72**	0.00417	0.19500	x	x			1.73	1.82	1.62
Bt.8107.1.S1_at	SP140	SP140 nuclear body protein	**1.72**	0.00054	0.16500					1.72	1.76	1.68
Bt.344.1.S1_at	HLA-DMA	major histocompatibility complex, class II, DM alpha	**1.71**	0.00445	0.19500			x		1.59	1.78	1.76
Bt.16018.1.S1_a_at	CASP4	caspase 4, apoptosis-related cysteine protease	**1.62**	0.00185	0.18300	x	x		x	1.60	1.69	1.58
Bt.23098.1.S1_at	QSCN6	quiescin Q6	**1.58**	0.00215	0.18300					1.51	1.60	1.62
Bt.22511.1.S1_at	UBE3B	ubiquitin protein ligase E3B	**1.55**	0.00296	0.18300					1.50	1.62	1.52
Bt.248.1.S1_at	CD38	CD38 antigen	**1.50**	0.00368	0.19400		x			1.50	1.44	1.57
**Priming decreased expression of:**												
Bt.21772.1.A1_at	ELTD1	EGF, latrophilin and seven transmembrane domain containing 1	**-3.49**	0.00220	0.18300					-3.11	-3.65	-3.75
Bt.8238.1.A1_at	TRIB3	tribbles homolog 3	**-2.11**	0.00288	0.18300					-1.97	-2.26	-2.10
Bt.22339.1.A1_at	TBXAS1	thromboxane A synthase 1	**-2.02**	0.00145	0.18300					-2.13	-1.99	-1.95
Bt.4509.1.S1_at	RASSF4	Ras association domain family 4	**-1.97**	0.00271	0.18300					-1.85	-2.08	-2.00
Bt.3520.1.S1_at	PYCR1	pyrroline-5-carboxylate reductase 1	**-1.89**	0.00158	0.18300					-1.80	-1.94	-1.94
Bt.4137.1.A1_at	G0S2	putative lymphocyte G0/G1 switch gene	**-1.88**	0.00396	0.19500	x	x			-1.89	-1.74	-2.00
Bt.16029.1.S3_at	FUT1	fucosyltransferase 1	**-1.80**	0.00420	0.19500					-1.68	-1.81	-1.92
Bt.10111.1.S1_at	FAM129A	family with sequence similarity 129, member A	**-1.77**	0.00299	0.18300					-1.79	-1.67	-1.86
Bt.26598.1.S1_at	PCTP	phosphatidylcholine transfer protein	**-1.63**	0.00306	0.18400					-1.67	-1.67	-1.54
Bt.13588.2.S1_at	PSAT1	phosphoserine aminotransferase 1	**-1.60**	0.00481	0.19500					-1.60	-1.51	-1.69

* Expression of the respective gene is known to be regulated by IL1, TNF, IL6, and / or IRF7 (based on Ingenuity Knowledge Database).

Table S1: All DEG from comparison Priming (P.) *versus* Control (C.)
B) Long time waiting experiment (13 IPA mapped DEG)

Probe set	Gene symbol	Description	Mean fold change (P./C.)	Parametric *p* -value	FDR	Expression regulated by (IPA)*				fold change of preparation:		
						**IL1** (4)	**TNF** (4)	**IL6** (3)	**IRF7** (1)	**1**	**2**	**3**
**Priming enhanced expression of:**												
Bt.552.1.S1_at	CCL5	chemokine (C-C motif) ligand 5	**63.03**	0.00299	0.25100	x	x	x	x	98.50	54.19	46.92
Bt.13628.2.S1_a_at	TGM3	transglutaminase 3	**6.77**	0.00211	0.24300					6.00	6.44	8.04
Bt.3686.1.S1_at	IL6	interleukin 6 (interferon, beta 2)	**5.50**	0.00043	0.21700	x	x	x		5.13	5.63	5.76
Bt.7165.1.S1_at	CXCL6	chemokine ligand 6	**3.87**	0.00283	0.25000	x	x	x		4.33	3.38	3.96
Bt.4802.1.S1_at	LTF	lactotransferrin	**1.87**	0.00382	0.26100					2.01	1.75	1.86
Bt.10398.1.S1_at	PTX3	pentaxin-related gene, rapidly induced by IL-1 beta	**1.67**	0.00226	0.24300	x	x			1.66	1.74	1.60
Bt.11679.1.S1_at	OLFML2A	olfactomedin-like 2A	**1.61**	0.00351	0.25100					1.58	1.55	1.70
Bt.21518.1.S1_at	SNF1LK	SNF1-like kinase	**1.58**	0.00239	0.24300					1.63	1.51	1.59
Bt.7122.1.S1_at	HELZ	helicase with zinc finger domain	**1.56**	0.00356	0.25100					1.52	1.52	1.65
**Priming decreased expression of:**												
Bt.13880.2.S1_at	DKK1	dickkopf homolog 1 (Xenopus laevis)	**-1.83**	0.00054	0.21700					-1.87	-1.78	-1.83
Bt.9640.1.A1_at	SPON1	spondin 1, extracellular matrix protein	**-1.75**	0.00354	0.25100					-1.83	-1.64	-1.80
Bt.3945.1.S1_at	TANC2	tetratricopeptide repeat, ankyrin repeat and coiled-coil containing 2	**-1.61**	0.00211	0.24300					-1.69	-1.57	-1.59
Bt.16808.2.A1_at	RASL12	RAS-like, family 12	**-1.60**	0.00048	0.21700					-1.58	-1.63	-1.60

* Expression of the respective gene is known to be regulated by IL1, TNF, IL6, and / or IRF7 (based on Ingenuity Knowledge Database).
