# Supplementary material for: Lipopolysaccharide priming enhances expression of effectors of immune defence while decreasing expression of pro-inflammatory cytokines in mammary epithelia cells from cows
Source: BMC Genomics. 2012 Jan 12;13:17. doi: 10.1186/1471-2164-13-17 (PMC3315725; doi:10.1186/1471-2164-13-17)
Supplement: Additional file 2 — Table S2: All DEG from comparison Induction post Priming (I.p.P.) versus Induction (I.). A) Short time waiting experiment (226 IPA mapped DEG). B) Long time waiting experiment (6 IPA mapped DEG) [file 1471-2164-13-17-S2.PDF]

Table S2: All DEG from comparison Induction post Priming (I.p.P.) versus Induction (I.)
A) Short time waiting experiment (226 IPA mapped DEG)

Probe set	Gene symbol	Description	I.p.P. *vs.* I.			Mean fold change compared to un-stimulated control (C.) and *p*-values						Expression regulated by (IPA)****				I.p.P. / I. Log ratio of preparation:		
			Mean fold change	Parametric *p*-value	FDR	I.p.P.	*p*	I.	*p*	P.	*p*	IL1 (44)	TNF (60)	IL6 (27)	IRF7 (34)	1	2	3
**Priming provokes a higher expression level after an *E. coli* challenge of:**																		
Bt.351.1.S1_at	**CORO1A***	coronin, actin binding protein, 1A	2.8**	0.00071	0.01320	1.6***	0.204	-1.7	0.021	1.6	0.042					1.01	1.57	1.83
Bt.24417.2.S1_at	**CD55**	CD55 molecule, decay accelerating factor for complement	2.6	0.00446	0.03490	1.6	0.028	-1.6	0.304	1.7	0.040	x	x			0.64	1.61	1.81
Bt.4751.1.S1_a_at	**HLA-DQA1**	major histocompatibility complex, class II, DQ alpha 1	2.3	0.00009	0.00516	3.7	0.009	1.6	0.126	3.8	0.007			x		1.26	1.02	1.37
Bt.13628.1.S1_at	TGM3	transglutaminase 3 (E polypeptide, protein-glutamine-gamma-glutamyltransferase)	2.3	0.00002	0.00324	4.4	0.001	1.9	0.011	2.2	0.003					1.30	1.19	1.13
Bt.15484.2.A1_at	**SLPI**	secretory leukocyte peptidase inhibitor	2.2	0.00085	0.01370	5.1	0.059	2.3	0.106	4.0	0.056		x			1.06	1.50	0.86
Bt.9683.1.A1_at	LY6G6E	lymphocyte antigen 6 complex, locus G6E	2.2	0.00292	0.02720	2.2	0.060	NC		2.1	0.038					0.96	0.78	1.65
Bt.9699.1.S1_at	CYP26A1	cytochrome P450, family 26, subfamily A, polypeptide 1	2.1	0.00054	0.01130	NC		-1.9	0.002	2.1	0.098					0.84	1.38	1.06
Bt.571.1.S1_at	**DEFB4**	defensin, beta 4	2.1	0.00275	0.02620	47.2	0.008	22.2	0.003	23.5	0.021	x	x			0.60	1.23	1.43
Bt.8552.1.S1_at	**HLA-DRA**	major histocompatibility complex, class II, DR alpha	2.1	0.00152	0.01850	5.8	0.006	2.8	0.002	6.4	0.003	x				0.75	1.00	1.47
Bt.22301.1.S1_at	ATP6V0A4	ATPase, H+ transporting, lysosomal V0 subunit a isoform 4	2.0	0.00031	0.00870	NC		-2.2	0.016	NC						1.00	0.82	1.24
Bt.24673.1.A1_at	LIPM	lipase, family member M	1.9	0.00082	0.01370	NC		-2.5	0.020	NC						0.72	0.87	1.22
Bt.9561.1.S1_at	**MUC1**	mucin 1, cell surface associated	1.8	0.00011	0.00566	11.4	0.000	6.2	0.000	5.4	0.001	x	x			0.99	0.79	0.84
Bt.10941.1.S1_at	MBNL3	muscleblind-like 3	1.8	0.00404	0.03260	NC		NC		2.2	0.016					0.54	0.80	1.28
Bt.2039.1.S1_at	CYP2S1	cytochrome P450, family 2, subfamily S, polypeptide 1	1.8	0.00036	0.00923	NC		NC		NC						0.97	0.65	0.92
Bt.350.1.S1_at	**HLA-DQB1**	major histocompatibility complex, class II, DQ beta 1	1.8	0.00457	0.03560	2.3	0.014	NC		2.2	0.058					0.44	0.84	1.16
Bt.7.1.S1_at	KRT6A	keratin 6A	1.7	0.00184	0.02070	2.7	0.013	1.6	0.002	NC			x			0.51	1.03	0.77
Bt.28282.1.S1_at	TFCP2L1	transcription factor CP2-like 1	1.7	0.00483	0.03690	-1.7	0.036	-2.8	0.017	NC						0.50	0.63	1.14
Bt.21137.1.S1_at	SLC34A2	solute carrier family 34, member 2	1.7	0.00238	0.02410	NC		NC		1.6	0.069					0.73	1.00	0.47
Bt.22699.3.S1_at	NFIA	nuclear factor I/A	1.7	0.00028	0.00822	NC		-1.7	0.011	NC						0.65	0.85	0.67
Bt.4778.1.S1_at	**SCNN1A**	sodium channel, nonvoltage-gated 1 alpha	1.6	0.00057	0.01160	NC		-1.6	0.002	NC		x	x	x		0.89	0.65	0.61
Bt.27318.1.S1_at	SMARCA1	SWI/SNF related, matrix associated, actin dependent regulator of chromatin, subfamily a, member 1	1.6	0.00098	0.01480	NC		-2.4	0.078	NC						0.87	0.77	0.51
Bt.24901.1.A1_at	NAP1L5	nucleosome assembly protein 1-like 5	1.6	0.00348	0.03000	NC		-1.7	0.033	NC						0.53	1.04	0.56
Bt.736.1.A1_at	**TGM1**	transglutaminase 1	1.6	0.00016	0.00624	3.0	0.001	1.8	0.008	NC		x				0.71	0.64	0.75
Bt.7478.1.S1_at	HSPB6	heat shock protein, alpha-crystallin-related, B6	1.6	0.00158	0.01900	-1.8	0.072	-2.9	0.009	NC						0.58	0.95	0.57
Bt.28068.1.S1_at	SLC5A1	solute carrier family 5 (sodium/glucose cotransporter), member 1	1.6	0.00026	0.00800	3.3	0.004	2.1	0.010	2.4	0.001					0.76	0.58	0.71
Bt.9779.1.S1_at	IFI27L2	interferon, alpha-inducible protein 27-like 2	1.6	0.00099	0.01480	11.6	0.000	7.3	0.001	8.7	0.001					0.62	0.53	0.86
Bt.3735.1.A1_at	MLLT11	myeloid/lymphoid or mixed-lineage leukemia (trithorax homolog, Drosophila); translocated to, 11	1.6	0.00151	0.01850	NC		NC		NC						0.44	0.80	0.75
Bt.28956.1.A1_at	RIMKLB	ribosomal modification protein rimK-like family member B	1.6	0.00045	0.01000	-1.8	0.016	-2.9	0.010	NC						0.69	0.53	0.75
Bt.22325.2.A1_at	CRCP	CGRP receptor component	1.6	0.00073	0.01330	NC		-1.6	0.178	NC			x			0.67	0.80	0.51
Bt.22409.1.S1_at	HSPA12A	heat shock 70kDa protein 12A	1.6	0.00325	0.02880	NC		-1.5	0.005	NC						0.85	0.37	0.70
Bt.12216.1.S1_at	C1QTNF5	C1q and tumor necrosis factor related protein 5	1.6	0.00020	0.00695	NC		NC		NC						0.61	0.68	0.62
Bt.25117.1.A1_at	MRAP2	melanocortin 2 receptor accessory protein 2	1.6	0.00254	0.02500	NC		-1.6	0.055	NC						0.40	0.67	0.84
Bt.20329.2.S1_at	ARL4D	ADP-ribosylation factor-like 4D	1.5	0.00019	0.00695	2.0	0.017	NC		NC						0.60	0.65	0.62
Bt.16951.1.A1_at	FZD2	frizzled homolog 2	1.5	0.00236	0.02410	NC		-1.7	0.002	NC						0.48	0.86	0.52
Bt.26958.1.A1_at	POLRMT	polymerase mitochondrial	1.5	0.00241	0.02440	NC		-1.7	0.084	NC						0.67	0.79	0.38
Bt.27202.1.A1_at	SOSTDC1	sclerostin domain containing 1	1.5	0.00427	0.03370	-2.5	0.057	-3.8	0.055	NC						0.76	0.32	0.70
Bt.11679.1.S1_at	OLFML2A	olfactomedin-like 2A	1.5	0.00022	0.00749	2.4	0.004	1.6	0.013	1.7	0.015					0.59	0.60	0.59
Bt.28395.1.S1_at	WDR54	WD repeat domain 54	1.5	0.00380	0.03160	NC		-1.6	0.022	NC						0.34	0.79	0.64
**Priming provokes a lower expression level after an *E. coli* challenge of:**																		
Bt.8479.1.A1_at	**NOS2**	nitric oxide synthase 2, inducible	-10.7	0.00014	0.00606	14.4	0.008	154.1	0.000	NC		x	x	x		-3.51	-2.71	-4.05
Bt.25303.1.A1_at	**GBP2**	guanylate binding protein 2, interferon-inducible	-8.1	0.00000	0.00215	35.6	0.000	287.1	0.000	9.7	0.007	x	x	x		-2.98	-2.91	-3.15
Bt.16070.2.S1_at	APOL3	apolipoprotein L, 3	-7.9	0.00043	0.00969	3.9	0.023	30.3	0.001	NC						-2.28	-2.83	-3.82
Bt.4856.1.S2_at	**IL1B**	interleukin 1, beta	-6.4	0.00133	0.01720	5.0	0.033	32.2	0.000	NC		x	x			-3.64	-2.58	-1.80
Bt.29044.1.S1_at	TIFA	TRAF-interacting protein with forkhead-associated domain	-6.0	0.00003	0.00371	6.1	0.004	36.3	0.001	NC						-2.98	-2.44	-2.31
Bt.24855.1.S1_at	**TNFSF13B**	tumor necrosis factor superfamily, member 13b	-5.8	0.00001	0.00293	15.4	0.001	89.4	0.001	NC			x		x	-2.78	-2.44	-2.39
Bt.16326.1.S1_at	TMEM140	transmembrane protein 140	-5.8	0.00002	0.00324	1.9	0.056	11.2	0.001	NC						-2.18	-2.75	-2.68
Bt.18440.2.S1_at	**GBP4**	guanylate binding protein 4	-5.8	0.00000	0.00215	NC		6.1	0.003	NC			x		x	-2.49	-2.39	-2.72
Bt.17081.2.S1_at	LMO2	LIM domain only 2	-5.3	0.00003	0.00342	NC		5.2	0.002	NC						-2.55	-2.04	-2.59
Bt.24813.2.S1_at	**RTP4**	receptor (chemosensory) transporter protein 4	-4.7	0.00049	0.01050	15.2	0.010	71.8	0.000	4.4	0.038				x	-2.49	-2.67	-1.55
Bt.16070.1.S1_at	CADPS	Ca++-dependent secretion activator	-4.3	0.00000	0.00215	11.3	0.001	48.2	0.001	1.5	0.319					-2.10	-2.11	-2.07
Bt.20790.1.S1_at	**TNFSF10**	tumor necrosis factor superfamily, member 10	-4.2	0.00004	0.00389	5.0	0.004	21.1	0.001	1.6	0.010	x	x		x	-1.95	-2.41	-1.89
Bt.18440.1.S1_at	**GBP6**	guanylate binding protein family, member 6	-4.2	0.00001	0.00293	NC		4.9	0.002	NC		x	x			-2.18	-1.84	-2.21
Bt.24795.1.A1_at	**IFIT2**	interferon-induced protein with tetratricopeptide repeats 2	-4.2	0.00005	0.00401	27.1	0.010	113.8	0.002	2.4	0.238			x	x	-2.40	-1.97	-1.84

Bt.8143.1.S1_at	**MX2**	myxovirus (influenza virus) resistance 2	**-4.2**	0.00001	0.00293	84.1	0.012	349.9	0.005	21.1	0.021				x	-1.90	-2.29	-1.99
Bt.17488.1.A1_at	**IL15**	interleukin 15	**-4.1**	0.00023	0.00749	NC		5.1	0.025	NC		x	x		x	-1.53	-2.23	-2.35
Bt.18873.1.A1_at	EPSTI1	epithelial stromal interaction 1	**-3.7**	0.00004	0.00372	1.7	0.001	6.1	0.004	NC						-2.04	-1.60	-2.00
Bt.7500.1.S1_at	RAB8B	RAB8B, member RAS oncogene family	**-3.5**	0.00001	0.00293	1.8	0.010	6.4	0.000	NC						-1.95	-1.63	-1.87
Bt.8997.1.S1_at	RANGAP1	Ran GTPase activating protein 1	**-3.4**	0.00001	0.00293	9.4	0.017	32.0	0.005	NC						-1.86	-1.83	-1.61
Bt.12553.1.S1_at	**HP**	haptoglobin	**-3.4**	0.00001	0.00293	56.7	0.000	191.9	0.000	14.3	0.002	x	x	x		-1.79	-1.60	-1.89
Bt.21102.1.S1_at	PARM1	prostate androgen-regulated mucin-like protein 1	**-3.4**	0.00002	0.00306	3.0	0.023	10.1	0.002	2.6	0.044					-1.95	-1.62	-1.71
Bt.1736.1.A1_at	**SOCS1**	suppressor of cytokine signaling 1	**-3.2**	0.00074	0.01330	1.6	0.091	5.0	0.020	NC		x	x	x	x	-1.24	-1.61	-2.21
Bt.29006.1.S1_at	**CYP2J2**	cytochrome P450, family 2, subfamily J, polypeptide 2	**-3.2**	0.00079	0.01370	1.9	0.092	6.0	0.000	NC						-1.78	-1.14	-2.12
Bt.26426.1.A1_at	RBM43	RNA binding motif protein 43	**-3.0**	0.00005	0.00413	3.4	0.000	10.3	0.002	1.6	0.095					-1.63	-1.37	-1.81
Bt.28704.1.S1_at	PLSCR4	phospholipid scramblase 4	**-3.0**	0.00208	0.02220	NC		2.4	0.019	NC						-2.19	-0.98	-1.59
Bt.23640.1.A1_a_at	**TLR3**	toll-like receptor 3	**-2.9**	0.00475	0.03660	NC		3.5	0.029	NC		x	x	x		-0.72	-1.89	-2.07
Bt.17195.1.A1_at	**EIF4E**	eukaryotic translation initiation factor 4E	**-2.9**	0.00005	0.00413	28.7	0.010	84.2	0.006	4.7	0.031	x				-1.38	-1.49	-1.79
Bt.22204.1.A1_at	ATP6V0A4	ATPase, H+ transporting, lysosomal V0 subunit a4	**-2.9**	0.00001	0.00293	4.9	0.004	14.1	0.003	NC						-1.44	-1.47	-1.66
Bt.26131.1.S1_at	CDADC1	cytidine and dCMP deaminase domain containing 1	**-2.9**	0.00002	0.00324	NC		3.8	0.014	NC						-1.43	-1.69	-1.43
Bt.21851.1.A1_at	**SP100**	nuclear antigen Sp100	**-2.8**	0.00303	0.02750	7.3	0.014	20.3	0.000	1.6	0.332					-1.28	-0.99	-2.18
Bt.15615.1.S1_at	**NLRC5**	NLR family, CARD domain containing 5	**-2.7**	0.00004	0.00394	2.3	0.000	6.3	0.002	NC						-1.51	-1.26	-1.60
Bt.24098.1.A1_at	**IFIH1**	interferon induced with helicase C domain 1	**-2.7**	0.00001	0.00293	7.3	0.023	19.8	0.011	2.7	0.036		x		x	-1.48	-1.40	-1.41
Bt.9217.1.A1_at	**HLA-A**	major histocompatibility complex, class I, A	**-2.7**	0.00007	0.00503	1.7	0.027	4.5	0.012	NC						-1.31	-1.28	-1.65
Bt.23233.1.S1_at	**USP18**	ubiquitin specific peptidase 18	**-2.6**	0.00033	0.00896	6.8	0.028	17.3	0.005	3.6	0.023				x	-1.11	-1.70	-1.25
Bt.1369.1.S1_at	SLC39A2	solute carrier family 39, member 2	**-2.5**	0.00014	0.00617	6.3	0.035	15.9	0.014	2.3	0.260					-1.58	-1.11	-1.31
Bt.20300.1.A1_at	TRANK1	tetratricopeptide repeat and ankyrin repeat containing 1	**-2.5**	0.00151	0.01850	8.9	0.042	22.2	0.009	3.5	0.130					-0.88	-1.79	-1.28
Bt.16725.2.A1_at	**UBE2L6**	ubiquitin-conjugating enzyme E2L 6	**-2.5**	0.00001	0.00293	2.7	0.012	6.6	0.005	NC					x	-1.35	-1.22	-1.32
Bt.24467.1.S1_at	**RSAD2**	radical S-adenosyl methionine domain containing 2	**-2.4**	0.00001	0.00293	154.7	0.034	373.3	0.026	18.6	0.094	x			x	-1.35	-1.26	-1.21
Bt.15788.3.S1_at	SLC27A1	solute carrier family 27 (fatty acid transporter), member 1	**-2.4**	0.00012	0.00576	27.6	0.024	65.9	0.011	7.8	0.042		x			-1.12	-1.49	-1.16
Bt.28313.1.S1_at	APOL2	apolipoprotein L, 2	**-2.4**	0.00009	0.00516	NC		2.9	0.001	NC						-1.44	-1.15	-1.11
Bt.22498.1.A1_at	DYNLT3	dynein, light chain, Tctex-type 3	**-2.3**	0.00010	0.00530	2.9	0.009	6.8	0.004	NC						-1.43	-1.12	-1.10
Bt.14676.1.S1_at	PARP14	poly (ADP-ribose) polymerase family, member 14	**-2.3**	0.00002	0.00293	3.0	0.022	7.0	0.005	1.9	0.033					-1.17	-1.29	-1.20
Bt.22980.1.S1_at	**TRIM21**	tripartite motif-containing 21	**-2.3**	0.00002	0.00324	2.0	0.008	4.6	0.000	NC					x	-1.27	-1.26	-1.12
Bt.16983.1.S1_at	**TRIM5**	tripartite motif-containing 5	**-2.3**	0.00003	0.00342	2.1	0.006	4.9	0.002	NC					x	-1.33	-1.14	-1.16
Bt.8945.1.S1_at	**TLR2**	toll-like receptor 2	**-2.3**	0.00002	0.00306	9.6	0.010	22.0	0.004	NC		x	x	x		-1.16	-1.17	-1.29
Bt.9296.1.A1_at	**GBP3**	guanylate binding protein 3	**-2.3**	0.00002	0.00324	3.4	0.006	7.7	0.001	2.6	0.008				x	-1.18	-1.29	-1.12
Bt.28908.1.A1_at	ZNF452	zinc finger protein 452	**-2.3**	0.00008	0.00503	3.7	0.005	8.5	0.002	1.7	0.031					-1.03	-1.36	-1.19
Bt.28094.1.S1_at	**CASP7**	caspase 7, apoptosis-related cysteine peptidase	**-2.3**	0.00001	0.00293	2.2	0.003	5.1	0.000	NC			x	x		-1.19	-1.16	-1.24
Bt.4509.1.S1_at	RASSF4	Ras association domain family 4	**-2.3**	0.00022	0.00749	-2.2	0.015	NC		-2.0	0.003					-1.44	-1.00	-1.10
Bt.18790.1.A1_at	**ADAR**	adenosine deaminase, RNA-specific	**-2.3**	0.00152	0.01850	2.0	0.073	4.5	0.003	1.7	0.016				x	-1.08	-1.62	-0.83
Bt.24012.1.A1_at	**GBP1**	guanylate binding protein 1, interferon-inducible, 67kDa	**-2.2**	0.00018	0.00674	3.0	0.011	6.7	0.008	NC		x	x	x	x	-1.25	-0.91	-1.30
Bt.2849.1.S1_at	SLC7A11	solute carrier family 7, (cationic amino acid transporter, y+ system) member 11	**-2.2**	0.00009	0.00518	-2.0	0.001	NC		-2.1	0.008					-1.28	-0.97	-1.20
Bt.13789.1.A1_at	**CD14**	CD14 antigen	**-2.2**	0.00193	0.02130	1.9	0.068	4.3	0.000	NC		x	x			-1.41	-0.66	-1.35
Bt.26665.2.S1_at	PLEKHA4	pleckstrin homology domain containing, family A (phosphoinositide binding specific) member 4	**-2.2**	0.00009	0.00524	1.6	0.116	3.4	0.012	NC						-1.31	-0.99	-1.12
Bt.28116.1.S1_at	TRIM16	tripartite motif-containing 16	**-2.2**	0.00009	0.00516	1.5	0.080	3.3	0.012	NC						-1.21	-1.25	-0.96
Bt.27943.1.S1_at	**STAT2**	signal transducer and activator of transcription 2, 113kDa	**-2.2**	0.00042	0.00964	NC		2.7	0.013	NC					x	-1.21	-0.84	-1.35
Bt.8917.1.S1_at	ALOX12	arachidonate lipoxygenase, epidermal	**-2.2**	0.00072	0.01320	-1.7	0.155	NC		NC						-1.24	-1.37	-0.78
Bt.1220.1.S1_at	SLFN11	schlafen family member 11	**-2.2**	0.00002	0.00293	4.1	0.015	9.0	0.006	2.8	0.015					-1.09	-1.15	-1.15
Bt.20017.1.S1_at	MLKL	mixed lineage kinase domain-like	**-2.2**	0.00042	0.00969	NC		2.6	0.003	NC						-1.42	-1.05	-0.90
Bt.17181.1.A1_at	PI15	protease inhibitor 15	**-2.2**	0.00323	0.02870	-2.6	0.020	NC		NC						-0.77	-1.64	-0.92
Bt.19329.1.A1_at	EFHD1	EF hand domain containing 1	**-2.1**	0.00107	0.01540	-3.8	0.004	-1.8	0.014	-2.1	0.009					-1.43	-1.02	-0.78
Bt.4714.1.S1_at	**MMP9**	matrix metallopeptidase 9	**-2.1**	0.00027	0.00815	5.4	0.023	11.3	0.013	1.9	0.143	x	x	x		-1.08	-1.29	-0.85
Bt.20517.1.S1_at	SLC25A28	solute carrier family 25, member 28	**-2.1**	0.00009	0.00516	1.7	0.036	3.5	0.010	NC						-1.23	-1.00	-0.98
Bt.12732.1.S1_at	NUB1	negative regulator of ubiquitin-like proteins 1	**-2.1**	0.00016	0.00624	NC		2.9	0.002	NC						-1.27	-0.97	-0.95
Bt.27564.1.A1_at	**DHX58**	DEXH (Asp-Glu-X-His) box polypeptide 58	**-2.1**	0.00008	0.00503	7.4	0.026	15.4	0.010	3.5	0.047				x	-0.92	-1.19	-1.07
Bt.18643.1.A1_at	**CFLAR**	CASP8 and FADD-like apoptosis regulator	**-2.1**	0.00010	0.00530	2.2	0.014	4.5	0.001	NC		x	x	x		-1.22	-0.98	-0.96
Bt.17229.1.A1_at	ZNFX1	zinc finger, NFX1-type containing 1	**-2.1**	0.00172	0.02030	2.7	0.057	5.6	0.004	1.5	0.039					-0.67	-1.40	-1.09
Bt.24033.1.A1_at	**DDX58**	DEAD (Asp-Glu-Ala-Asp) box polypeptide 58	**-2.1**	0.00004	0.00371	4.6	0.020	9.4	0.007	3.0	0.033		x		x	-0.96	-1.11	-1.06
Bt.12440.1.A1_at	**IGFBP5**	insulin-like growth factor binding protein 5	**-2.1**	0.00037	0.00926	-3.6	0.033	-1.8	0.061	NC		x		x		-1.29	-0.86	-0.96
Bt.24663.1.A1_at	FOXS1	forkhead box S1	**-2.1**	0.00255	0.02510	NC		2.1	0.047	NC						-0.71	-0.91	-1.49
Bt.18415.1.A1_at	FTSJD1	FtsJ methyltransferase domain containing 1	**-2.0**	0.00027	0.00805	2.7	0.013	5.5	0.012	NC						-1.05	-0.80	-1.19
Bt.21803.1.A1_at	NT5C3	5'-nucleotidase, cytosolic III	**-2.0**	0.00028	0.00822	NC		2.5	0.009	NC						-1.15	-0.78	-1.11
Bt.14047.1.A1_at	TRIM6	tripartite motif-containing 6	**-2.0**	0.00004	0.00380	1.9	0.000	3.8	0.001	NC						-1.06	-0.93	-1.04
Bt.17729.1.A1_at	**IFI44**	interferon-induced protein 44	**-2.0**	0.00039	0.00933	20.3	0.013	40.8	0.007	14.0	0.008				x	-1.06	-1.20	-0.76
Bt.24665.1.S1_at	**CIITA**	class II, major histocompatibility complex, transactivator	**-2.0**	0.00184	0.02070	NC		2.7	0.004	NC		x	x	x		-0.70	-0.92	-1.39
Bt.22130.1.S1_at	IFIT5	interferon-induced protein with tetratricopeptide repeats 5	**-2.0**	0.00004	0.00401	3.4	0.002	6.9	0.002	1.9	0.015		x			-1.03	-0.91	-1.06
Bt.15788.1.S1_a_at	BST2	bone marrow stromal cell antigen 2	**-2.0**	0.00003	0.00360	20.5	0.012	40.9	0.008	7.0	0.026		x			-1.03	-1.03	-0.94

Bt.26268.1.A1_at	**MMP13**	matrix metallopeptidase 13 (collagenase 3)	-2.0	0.00076	0.01350	NC		2.0	0.027	NC		x	x	x		-0.82	-1.31	-0.86
Bt.21835.1.S1_at	PKIG	protein kinase inhibitor gamma	-2.0	0.00024	0.00771	NC		2.0	0.002	NC						-1.21	-0.92	-0.86
Bt.6833.1.S1_at	PAPD4	PAP associated domain containing 4	-2.0	0.00023	0.00755	1.5	0.013	3.0	0.002	NC						-1.17	-0.81	-0.98
Bt.24933.1.S1_at	RARRES1	retinoic acid receptor responder 1	-2.0	0.00099	0.01480	-4.2	0.024	-2.1	0.151	-2.3	0.015					-1.03	-1.24	-0.67
Bt.9030.1.S1_at	**TLR4**	toll-like receptor 4	-2.0	0.00045	0.01000	1.6	0.053	3.1	0.000	NC		x	x	x	x	-1.23	-0.81	-0.89
Bt.27071.1.S1_at	TRIM38	tripartite motif-containing 38	-1.9	0.00003	0.00364	NC		2.6	0.000	NC						-0.98	-0.96	-0.92
Bt.16350.1.S1_at	**GBP5**	guanylate binding protein 5	-1.9	0.00471	0.03640	NC		2.5	0.009	NC					x	-1.37	-0.98	-0.50
Bt.5768.2.S1_at	**IRF7**	interferon regulatory factor 7	-1.9	0.00050	0.01050	1.8	0.080	3.5	0.008	NC		x	x			-0.79	-1.20	-0.84
Bt.9745.1.A1_at	KRT13	keratin 13	-1.9	0.00030	0.00850	-3.6	0.000	-1.9	0.013	-2.2	0.020					-0.75	-0.95	-1.12
Bt.22116.1.A1_at	IL18BP	interleukin 18 binding protein	-1.9	0.00005	0.00412	34.6	0.004	66.4	0.003	11.5	0.005		x			-1.01	-0.91	-0.89
Bt.3247.1.S1_at	MATN2	matrilin 2	-1.9	0.00122	0.01660	-5.5	0.007	-2.9	0.007	-3.0	0.022					-1.08	-0.60	-1.13
Bt.26606.1.S1_at	CHST1	carbohydrate sulfotransferase 1	-1.9	0.00129	0.01720	NC		1.9	0.022	NC						-0.64	-1.23	-0.95
Bt.9560.1.S1_at	**CCL20**	chemokine (C-C motif) ligand 20	-1.9	0.00016	0.00624	219.6	0.000	417.8	0.000	NC		x	x	x		-1.07	-0.79	-0.92
Bt.12250.1.S1_at	PPP2R3C	protein phosphatase 2 (formerly 2A), regulatory subunit B'', gamma	-1.9	0.00020	0.00695	NC		2.2	0.000	NC						-1.10	-0.81	-0.88
Bt.1929.1.S1_at	FAM46A	family with sequence similarity 46, member A	-1.9	0.00011	0.00545	NC		1.9	0.003	NC						-0.92	-1.04	-0.81
Bt.22021.1.S1_at	**IFI16**	interferon, gamma-inducible protein 16	-1.9	0.00005	0.00427	6.0	0.018	11.3	0.010	3.9	0.017				x	-0.96	-0.93	-0.84
Bt.9075.1.S1_at	COIL	coilin	-1.9	0.00007	0.00503	2.6	0.012	4.8	0.004	1.6	0.011					-0.82	-0.91	-0.99
Bt.11043.1.S1_a_at	BCL2L12	BCL2-like 12	-1.9	0.00115	0.01600	NC		2.0	0.001	NC						-1.22	-0.72	-0.78
Bt.1029.1.S1_at	NCOA7	nuclear receptor coactivator 7	-1.9	0.00080	0.01370	1.5	0.120	2.9	0.054	NC						-1.14	-0.65	-0.91
Bt.25103.1.S1_at	TDRD7	tudor domain containing 7	-1.9	0.00010	0.00530	2.3	0.017	4.3	0.003	1.6	0.019		x			-1.00	-0.90	-0.79
Bt.28523.1.S1_at	DTX3L	deltex 3-like (Drosophila)	-1.8	0.00005	0.00413	3.1	0.010	5.7	0.004	1.9	0.026					-0.93	-0.90	-0.83
Bt.13095.1.S1_at	HAS3	hyaluronan synthase 3	-1.8	0.00010	0.00530	NC		2.0	0.068	NC						-0.94	-0.95	-0.77
Bt.9792.1.S1_at	ARRDC4	arrestin domain containing 4	-1.8	0.00033	0.00896	NC		2.3	0.001	NC						-1.00	-0.68	-0.97
Bt.8107.1.S1_at	SP140	SP140 nuclear body protein	-1.8	0.00025	0.00784	2.0	0.001	3.7	0.001	1.7	0.001					-1.03	-0.72	-0.88
Bt.13844.3.A1_at	FBXO33	F-box protein 33	-1.8	0.00018	0.00674	2.0	0.003	3.7	0.005	NC						-0.74	-0.86	-1.01
Bt.13588.2.S1_at	PSAT1	phosphoserine aminotransferase 1	-1.8	0.00015	0.00624	-2.2	0.007	NC		-1.6	0.005					-0.85	-1.00	-0.77
Bt.10077.1.S3_at	**IRF1**	interferon regulatory factor 1	-1.8	0.00108	0.01550	1.8	0.040	3.3	0.005	NC		x	x	x	x	-1.01	-0.58	-1.03
Bt.4342.1.S1_at	SELP	selectin P	-1.8	0.00015	0.00624	NC		NC		NC			x			-0.98	-0.74	-0.87
Bt.22056.2.S1_a_at	ABHD1	abhydrolase domain containing 1	-1.8	0.00025	0.00794	2.0	0.081	3.6	0.013	2.4	0.001					-0.95	-0.94	-0.69
Bt.29506.1.S1_at	CCDC82	coiled-coil domain containing 82	-1.8	0.00014	0.00606	NC		1.9	0.002	NC						-0.97	-0.76	-0.82
Bt.3774.1.A1_at	RND1	Rho family GTPase 1	-1.8	0.00016	0.00633	12.4	0.005	22.3	0.002	NC			x			-0.85	-0.72	-0.96
Bt.9267.1.A1_at	APOBEC3B	apolipoprotein B mRNA editing enzyme, catalytic polypeptide-like 3B	-1.8	0.00335	0.02930	NC		1.9	0.017	NC			x			-1.21	-0.77	-0.54
Bt.12699.1.S1_at	PTPRU	protein tyrosine phosphatase, receptor type, U	-1.8	0.00424	0.03360	-1.6	0.043	NC		NC						-0.42	-0.97	-1.12
Bt.1543.1.A1_at	**NAMPT**	nicotinamide phosphoribosyltransferase	-1.8	0.00008	0.00503	NC		2.6	0.001	NC		x	x	x	x	-0.90	-0.78	-0.84
Bt.22144.2.S1_at	STARD4	StAR-related lipid transfer (START) domain containing 4	-1.8	0.00040	0.00946	NC		2.2	0.016	NC						-0.80	-1.02	-0.68
Bt.5540.1.S1_a_at	GTF2B	general transcription factor IIB	-1.8	0.00007	0.00503	2.3	0.002	4.1	0.001	NC						-0.80	-0.80	-0.88
Bt.19107.1.S1_at	VPS54	vacuolar protein sorting 54	-1.8	0.00010	0.00530	2.2	0.004	3.8	0.001	NC						-0.78	-0.78	-0.91
Bt.22773.2.S1_at	SLC25A19	solute carrier family 25, member 19	-1.7	0.00048	0.01040	1.5	0.092	2.7	0.037	NC						-0.61	-0.92	-0.89
Bt.13556.2.A1_x_at	CFH	complement factor H	-1.7	0.00110	0.01560	-2.0	0.039	NC		NC						-0.93	-0.53	-0.91
Bt.16946.1.A1_at	CUL4B	cullin 4B	-1.7	0.00008	0.00503	1.5	0.009	2.6	0.003	1.6	0.076					-0.82	-0.81	-0.75
Bt.22275.1.A1_at	**ISG20**	interferon stimulated exonuclease gene 20kDa	-1.7	0.00025	0.00784	4.2	0.005	7.3	0.001	1.9	0.009				x	-0.88	-0.84	-0.64
Bt.13880.1.S1_at	DKK1	dickkopf homolog 1	-1.7	0.00312	0.02790	1.5	0.216	2.6	0.026	NC						-1.07	-0.45	-0.84
Bt.3196.1.S1_at	STRA6	stimulated by retinoic acid gene 6 homolog	-1.7	0.00216	0.02270	NC		2.1	0.011	NC						-0.56	-1.09	-0.68
Bt.17036.1.A1_s_at	WARS	tryptophanyl-tRNA synthetase	-1.7	0.00113	0.01590	NC		1.8	0.017	NC				x		-0.61	-1.03	-0.68
Bt.20922.1.S1_at	**OAS1**	2',5'-oligoadenylate synthetase 1, 40/46kDa	-1.7	0.00079	0.01370	33.8	0.026	57.9	0.015	18.0	0.029		x		x	-0.63	-1.00	-0.70
Bt.24484.2.S1_a_at	CLEC12A	C-type lectin domain family 12, member A	-1.7	0.00055	0.01130	NC		2.4	0.003	NC						-0.90	-0.58	-0.84
Bt.22219.1.S1_at	JHDM1D	jumonji C domain containing histone demethylase 1 homolog D (S. cerevisiae)	-1.7	0.00013	0.00606	1.6	0.035	2.7	0.008	NC						-0.86	-0.70	-0.76
Bt.20054.1.S1_at	**IRF9**	interferon regulatory factor 9	-1.7	0.00011	0.00566	2.2	0.013	3.8	0.008	1.7	0.047			x	x	-0.84	-0.72	-0.75
Bt.25111.1.A1_at	**IFI44L**	interferon-induced protein 44	-1.7	0.00043	0.00969	11.4	0.024	19.4	0.017	7.8	0.025				x	-0.88	-0.82	-0.59
Bt.4675.1.S1_a_at	**MX1**	myxovirus (influenza virus) resistance 1	-1.7	0.00050	0.01050	17.5	0.024	29.8	0.013	8.4	0.034	x	x		x	-0.73	-0.94	-0.62
Bt.22141.1.S1_at	JUP	junction plakoglobin	-1.7	0.00201	0.02160	1.7	0.032	2.9	0.020	NC						-0.46	-0.88	-0.94
Bt.5684.1.S1_at	PNPT1	polyribonucleotide nucleotidyltransferase 1	-1.7	0.00008	0.00503	1.9	0.003	3.2	0.001	NC						-0.77	-0.77	-0.74
Bt.27463.1.A1_at	HERC6	hect domain and RLD 6	-1.7	0.00030	0.00846	1.6	0.147	2.7	0.028	NC						-0.66	-0.90	-0.72
Bt.7751.1.S1_at	SYT4	synaptotagmin IV	-1.7	0.00014	0.00606	-1.9	0.008	NC		NC						-0.79	-0.81	-0.67
Bt.28624.1.S1_at	SAMD9	sterile alpha motif domain containing 9	-1.7	0.00057	0.01160	3.3	0.018	5.6	0.005	2.2	0.034					-0.56	-0.86	-0.83
Bt.20923.1.S1_at	**EIF2AK2**	eukaryotic translation initiation factor 2-alpha kinase 2	-1.7	0.00062	0.01210	2.6	0.012	4.3	0.001	2.0	0.010		x			-0.56	-0.88	-0.77
Bt.8053.3.S1_at	ATAD1	ATPase family, AAA domain containing 1	-1.7	0.00019	0.00695	NC		2.2	0.004	NC						-0.77	-0.80	-0.63
Bt.13130.1.S1_at	**CD40**	CD40 molecule, TNF receptor superfamily member 5	-1.7	0.00037	0.00933	3.6	0.009	5.9	0.002	NC		x	x	x	x	-0.84	-0.59	-0.77
Bt.10988.1.S1_at	USP25	ubiquitin specific protease 25	-1.7	0.00102	0.01510	NC		NC		NC						-0.89	-0.51	-0.78
Bt.29152.1.S1_at	TM4SF12	transmembrane 4 superfamily member 12	-1.7	0.00235	0.02400	-7.4	0.004	-4.5	0.001	-2.1	0.026					-1.01	-0.52	-0.63
Bt.13370.3.S1_at	RICTOR	RPTOR independent companion of MTOR, complex 2	-1.6	0.00019	0.00695	1.6	0.054	2.6	0.011	NC						-0.65	-0.81	-0.71
Bt.4818.1.S1_at	BEX2	brain expressed X-linked 2	-1.6	0.00016	0.00624	-2.0	0.008	NC		-1.9	0.007					-0.76	-0.77	-0.64
Bt.20069.1.S1_at	**DAXX**	death-domain associated protein	-1.6	0.00016	0.00630	NC		2.0	0.005	NC			x			-0.79	-0.68	-0.67

Bt.23178.1.S2_at	**DCN**	decorin	**-1.6**	0.00253	0.02490	NC		NC		NC		x	x	x		-0.88	-0.42	-0.85
Bt.22729.1.S1_at	HLA-L	major histocompatibility complex, class I, L, pseudogene	**-1.6**	0.00091	0.01410	NC		**2.4**	0.004	NC						-0.89	-0.53	-0.72
Bt.16340.1.A1_at	ARHGAP42	Rho GTPase activating protein 42	**-1.6**	0.00216	0.02270	NC		*2.1*	0.006	NC						-0.51	-0.64	-0.99
Bt.4645.1.S1_at	FAM129A	family with sequence similarity 129, member A	**-1.6**	0.00013	0.00606	*-2.1*	0.030	NC		NC						-0.66	-0.75	-0.72
Bt.5056.2.A1_at	EFEMP1	EGF-containing fibulin-like extracellular matrix protein 1	**-1.6**	0.00100	0.01490	*-2.7*	0.010	*-1.6*	0.032	*-1.9*	0.089					-0.51	-0.72	-0.89
Bt.16018.1.S2_at	**CASP4**	caspase 4, apoptosis-related cysteine peptidase	**-1.6**	0.00017	0.00645	**3.1**	0.000	**5.1**	0.001	**1.6**	0.001	x	x		x	-0.75	-0.63	-0.75
Bt.29894.1.S1_at	**PHF15**	PHD finger protein 15	**-1.6**	0.00067	0.01250	*2.0*	0.036	*3.3*	0.016	NC						-0.76	-0.52	-0.82
Bt.26598.1.S1_at	PCTP	phosphatidylcholine transfer protein	**-1.6**	0.00086	0.01370	**-2.8**	0.002	**-1.7**	0.002	**-1.6**	0.003					-0.88	-0.70	-0.53
Bt.19053.1.A1_at	USP34	ubiquitin specific protease 34	**-1.6**	0.00015	0.00624	NC		NC		NC						-0.74	-0.64	-0.71
Bt.20512.1.S1_at	COL15A1	collagen, type XV, alpha 1	**-1.6**	0.00302	0.02750	*-2.7*	0.034	*-1.6*	0.024	*-1.8*	0.153		x			-0.79	-0.40	-0.90
Bt.9570.1.S1_at	**TNFAIP2**	tumor necrosis factor, alpha-induced protein 2	**-1.6**	0.00151	0.01850	**5.9**	0.003	*9.5*	0.006	NC		x	x			-0.46	-0.86	-0.76
Bt.26400.1.A1_at	ELMO2	engulfment and cell motility 2	**-1.6**	0.00015	0.00624	NC		**1.9**	0.004	NC						-0.65	-0.66	-0.74
Bt.10962.2.S1_at	VCPIP1	valosin containing protein (p97)/p47 complex interacting protein 1	**-1.6**	0.00027	0.00803	*1.5*	0.021	**2.4**	0.004	NC						-0.58	-0.71	-0.75
Bt.2568.1.S1_at	PLIN2	perilipin 2	**-1.6**	0.00016	0.00633	NC		**2.3**	0.001	NC			x			-0.68	-0.63	-0.73
Bt.26851.1.S1_at	DRAM1	DNA-damage regulated autophagy modulator 1	**-1.6**	0.00021	0.00736	*1.7*	0.020	**2.8**	0.005	NC						-0.60	-0.68	-0.74
Bt.24539.1.S1_at	TMEM106A	transmembrane protein 106A	**-1.6**	0.00039	0.00933	NC		**2.4**	0.004	NC						-0.80	-0.62	-0.61
Bt.12849.2.S1_at	SLCO2B1	solute carrier organic anion transporter family, member 2B1	**-1.6**	0.00024	0.00775	NC		NC		NC						-0.58	-0.72	-0.72
Bt.13869.1.A1_at	TRIM25	tripartite motif-containing 25	**-1.6**	0.00024	0.00778	**2.3**	0.001	**3.6**	0.001	NC						-0.73	-0.68	-0.58
Bt.28411.1.S1_at	SLC16A6	solute carrier family 16, member 6	**-1.6**	0.00062	0.01210	NC		NC		NC						-0.67	-0.78	-0.51
Bt.422.1.S2_at	**IGFBP3**	insulin-like growth factor binding protein 3	**-1.6**	0.00081	0.01370	NC		*2.2*	0.008	NC		x	x	x		-0.50	-0.81	-0.65
Bt.22496.1.S1_at	CBR3	carbonyl reductase 3	**-1.6**	0.00345	0.03000	NC		**2.3**	0.001	NC						-0.94	-0.45	-0.57
Bt.3211.1.S2_at	GABARAPL1	GABA(A) receptor-associated protein like 1	**-1.6**	0.00016	0.00624	NC		**2.1**	0.003	NC						-0.65	-0.68	-0.63
Bt.22630.1.S1_at	DHDH	dihydrodiol dehydrogenase (dimeric)	**-1.6**	0.00039	0.00933	*-1.7*	0.010	NC		NC						-0.59	-0.59	-0.77
Bt.24039.1.A1_at	CCDC6	coiled-coil domain containing 6	**-1.6**	0.00040	0.00941	NC		*1.6*	0.015	NC						-0.61	-0.76	-0.57
Bt.3841.2.S1_at	**CD83**	CD83 antigen	**-1.6**	0.00264	0.02540	NC		*2.0*	0.009	NC		x	x	x		-0.79	-0.76	-0.38
Bt.26760.1.S1_at	ARRDC2	arrestin domain containing 2	**-1.6**	0.00038	0.00933	*2.3*	0.013	*3.6*	0.009	NC						-0.71	-0.68	-0.53
Bt.25663.1.A1_at	CPNE8	copine VIII	**-1.6**	0.00086	0.01370	NC		*1.9*	0.010	NC						-0.51	-0.60	-0.80
Bt.28164.2.S1_at	EFR3A	EFR3 homolog A (S. cerevisiae)	**-1.6**	0.00183	0.02070	NC		1.9	0.013	**1.6**	0.019					-0.85	-0.60	-0.45
Bt.5366.1.S1_at	PROS1	protein S	**-1.5**	0.00036	0.00923	*-3.0*	0.060	*-2.0*	0.140	*-1.7*	0.128					-0.60	-0.56	-0.73
Bt.23509.1.S1_at	PPAP2B	phosphatidic acid phosphatase type 2B	**-1.5**	0.00087	0.01380	*2.0*	0.030	*3.1*	0.006	NC						-0.79	-0.53	-0.56
Bt.25471.2.A1_at	ATXN3	ataxin 3	**-1.5**	0.00118	0.01640	NC		*1.7*	0.009	NC						-0.78	-0.63	-0.46
Bt.22506.2.A1_at	VIPR2	vasoactive intestinal peptide receptor 2	**-1.5**	0.00135	0.01730	NC		*2.0*	0.022	NC			x			-0.58	-0.81	-0.48
Bt.9391.2.S1_at	**BIRC3**	baculoviral IAP repeat-containing 3	**-1.5**	0.00023	0.00752	**6.1**	0.001	**9.5**	0.001	NC		x	x			-0.58	-0.67	-0.62
Bt.11221.1.S1_at	GYPC	glycophorin C	**-1.5**	0.00400	0.03260	**-5.7**	0.003	*-3.7*	0.022	*-2.1*	0.035					-0.74	-0.79	-0.33
Bt.9391.1.A1_at	**BIRC2**	baculoviral IAP repeat-containing 2	**-1.5**	0.00039	0.00933	**4.8**	0.000	**7.4**	0.001	NC		x	x			-0.66	-0.52	-0.68
Bt.19349.1.A1_at	GOLGA4	golgi autoantigen, golgin subfamily a, 4	**-1.5**	0.00471	0.03640	*3.5*	0.059	*5.4*	0.019	NC						-0.88	-0.61	-0.36
Bt.6586.1.S1_at	NOTCH3	Notch homolog 3	**-1.5**	0.00245	0.02460	NC		NC		NC						-0.40	-0.62	-0.82
Bt.13073.1.S1_at	**SERPINB2**	serine proteinase inhibitor, clade B, member 2	**-1.5**	0.00127	0.01690	*1.9*	0.041	**2.8**	0.004	NC		x	x			-0.75	-0.43	-0.66
Bt.11500.1.A1_at	INPP5B	inositol polyphosphate-5-phosphatase, 75kDa	**-1.5**	0.00020	0.00695	*-2.1*	0.027	NC		NC						-0.62	-0.62	-0.60
Bt.4816.1.S1_at	**ANGPTL4**	angiopoietin-like 4	**-1.5**	0.00027	0.00805	NC		**2.2**	0.001	NC		x	x			-0.57	-0.67	-0.59
Bt.24801.1.A1_at	BHLHB5	basic helix-loop-helix domain containing, class B, 5	**-1.5**	0.00029	0.00841	NC		*1.8*	0.091	NC						-0.67	-0.58	-0.56
Bt.8238.1.A1_at	TRIB3	tribbles homolog 3	**-1.5**	0.00074	0.01330	**-1.9**	0.003	NC		**-2.1**	0.003					-0.67	-0.46	-0.68
Bt.27957.1.A1_at	PARP8	poly polymerase family, member 8	**-1.5**	0.00325	0.02880	NC		NC		NC						-0.85	-0.54	-0.41
Bt.278.1.S1_at	**SAA3**	serum amyloid A3	**-1.5**	0.00054	0.01130	**268.7**	0.001	**405.3**	0.000	**146.1**	0.001	x				-0.51	-0.70	-0.57
Bt.2501.1.S1_at	**SOD2**	superoxide dismutase 2, mitochondrial	**-1.5**	0.00098	0.01480	**4.0**	0.000	**6.0**	0.001	NC		x	x	x		-0.71	-0.44	-0.62
Bt.18116.1.S1_at	PARP12	poly (ADP-ribose) polymerase family, member 12	**-1.5**	0.00261	0.02530	*1.8*	0.166	*2.8*	0.043	*1.8*	0.117					-0.70	-0.71	-0.36
Bt.12304.1.S1_at	**ISG15**	ISG15 ubiquitin-like modifier	**-1.5**	0.00062	0.01210	*11.5*	0.021	*17.3*	0.014	*7.4*	0.024	x	x		x	-0.61	-0.67	-0.47
Bt.28436.1.S1_at	FAM126B	family with sequence similarity 126, member B	**-1.5**	0.00031	0.00870	NC		**2.1**	0.003	NC						-0.53	-0.60	-0.63

* bold letters, part of a network connected by the IPA relationship "Expression" (Fig. 3B)
** bold numbers, fc>1.5, p<0.005
*** italic number, fc>1.5, p>0.005
NC, No change fc<1.5
grey, I.p.P. not changed compared to un-stimulated control
dark red, I.p.P. compared to un-stimulated control fc>1.5, p<0.005 (significant)
light red, I.p.P. compared to un-stimulated control fc>1.5, p>0.005 (not significant)
dark green, I.p.P. compared to un-stimulated control fc<-1.5, p<0.005 (significant)
light green, I.p.P. compared to un-stimulated control fc<-1.5, p>0.005 (not significant)
**** Expression of the respective gene is known to be regulated by IL1, TNF, IL6, and / or IRF7 (based on Ingenuity Knowledge Database).

Table S2: All DEG from comparison Induction post Priming (I.p.P.) *versus* Induction (I.)

B) Long time waiting experiment (6 IPA mapped DEG)

Probe set	Gene symbol	Description	I.p.P. *vs.* I. Mean fold change	Parametric *p*-value	FDR	Expression regulated by (IPA)* IL1 (0)	TNF (0)	IL6 (1)	IRF7 (0)	I.p.P. / I. Log ratio of preparation : 1	2	3
**Priming provokes a higher expression level after an *E. coli* challenge of:**												
Bt.9196.1.A1_at	MKI67	antigen identified by monoclonal antibody Ki-67	**1.89**	0.00471	0.47400			x		1.77	1.87	2.05
Bt.29431.1.A1_at	PMPCB	peptidase beta	**1.74**	0.00166	0.39200					1.80	1.76	1.67
**Priming provokes a lower expression level after an *E. coli* challenge of:**												
Bt.17510.1.A1_at	PAM	peptidylglycine alpha-amidating monooxygenase	**-2.2**	0.00435	0.47400					-1.99	-2.15	-2.38
Bt.24222.2.S1_at	WWTR1	WW domain containing transcription regulator 1	**-1.7**	0.00415	0.47400					-1.66	-1.82	-1.62
Bt.27957.1.A1_at	PARP8	poly polymerase family, member 8	**-1.7**	0.00251	0.43500					-1.61	-1.68	-1.76
Bt.21772.1.A1_at	ELTD1	EGF, latrophilin and seven transmembrane domain containing 1	**-1.6**	0.00134	0.35400					-1.65	-1.56	-1.64

* Expression of the respective gene is known to be regulated by IL1, TNF, IL6, and / or IRF7 (based on Ingenuity Knowledge Database).
