# Supplementary material for: Lipopolysaccharide priming enhances expression of effectors of immune defence while decreasing expression of pro-inflammatory cytokines in mammary epithelia cells from cows
Source: BMC Genomics. 2012 Jan 12;13:17. doi: 10.1186/1471-2164-13-17 (PMC3315725; doi:10.1186/1471-2164-13-17)
Supplement: Additional file 3 — Table S3: Comparison of RT-qPCR and microarray measurements of selected candidate genes [file 1471-2164-13-17-S3.PDF]

**Table S3: Comparison of qRT-PCR and microarray measurements of selected candidate genes.**

| | Gene | Short time waiting experiment | | | | Long time waiting experiment | | | |
|---|---|---|---|---|---|---|---|---|---|
| | | P. *vs.* C. | | I.p.P. *vs.* I. | | P. *vs.* C. | | I.p.P. *vs.* I. | |
| | | qRT-PCR | Microarray | qRT-PCR | Microarray | qRT-PCR | Microarray | qRT-PCR | Microarray |
| Proinflammatory master cytokine | **IL1B** | NC* | NC | **-2.0**** <br> *0.063* *** | **-6.4** <br> *0.001* | NC | NC | **-1.8** <br> *0.010* | NC |
| | **TNF-α** | NC | NC | **-1.6** <br> *0.093* | NC | **2.4** <br> *0.052* | NC | **-1.4** <br> *0.027* | NC |
| | **IL6** | NC | NC | **-1.5** <br> *0.045* | NC | **2.7** <br> *0.033* | **5.5** <br> *0.0004* | NC | NC |
| Effector driven by IL1B and/or IRF7 | **NOS2** | NC | NC | **-3.4** <br> *0.037* | **-10.7** <br> *0.0001* | NC | NC | **-1.5** <br> *0.028* | NC |
| | **IL15** | NC | NC | **-2.2** <br> *0.005* | **-4.1** <br> *0.0002* | NC | NC | NC | NC |
| | **MX2** | **5.7** <br> *0.028* | NC | **-4.1** <br> *0.011* | **-4.2** <br> *<0.0001* | NC | NC | NC | NC |
| | **RTP4** | **6.4** <br> *0.015* | **13.3** <br> *0.003* | **-2.3** <br> *0.005* | **-4.7** <br> *0.0005* | **3.0** <br> *0.028* | NC | NC | NC |
| Chemokine | **CCL5** | **22.0** <br> *0.023* | **466.5** <br> *0.0003* | NC | NC | **5.1** <br> *0.301* | **63.0** <br> *0.003* | NC | NC |
| | **IL8** | **2.5** <br> *0.008* | NC | NC | NC | **2.3** <br> *0.007* | NC | NC | NC |
| Anti-microbial | **LAP** | **8.7** <br> *<0.001* | NC | **1.6** <br> *0.017* | **2.1** <br> *0.003* | **1.7** <br> *0.026* | NC | NC | NC |
| | **SLPI** | **2.4** <br> *0.044* | NC | **1.7** <br> *0.011* | **2.2** <br> *0.0009* | **1.4** <br> *0.044* | NC | NC | NC |
| Cell and tissue protection | **TGM3** | **2.2** <br> *0.010* | **3.1** <br> *0.0001* | **2.1** <br> *0.002* | **2.3** <br> *<0.0001* | **3.3** <br> *0.022* | **6.8** <br> *0.002* | **2.7** <br> *0.044* | NC |
| | **SAA3** | **77.4** <br> *0.003* | **146.1** <br> *0.0009* | NC | **-1.5** <br> *0.001* | **4.8** <br> *<0.0001* | NC | NC | NC |
| | **LTF** | **3.9** <br> *0.012* | **5.1** <br> *0.0002* | NC | NC | **1.9** <br> *0.002* | **1.9** <br> *0.004* | NC | NC |

* NC, not changed
** bold numbers, fold changes
*** italic numbers, *p*-value, paired t-test
