# Supplementary material for: Lipopolysaccharide priming enhances expression of effectors of immune defence while decreasing expression of pro-inflammatory cytokines in mammary epithelia cells from cows
Source: BMC Genomics. 2012 Jan 12;13:17. doi: 10.1186/1471-2164-13-17 (PMC3315725; doi:10.1186/1471-2164-13-17)
Supplement: Additional file 4 — Table S4: All RT-qPCR values (relative mRNA copy numbers) contributing to Figure 4. [file 1471-2164-13-17-S4.PDF]

Table S4: All RT-qPCR values (relative mRNA copy numbers) contributing to Fig. 4

Treatment	MEC from animal	Gene													
		IL1B	TNF-α	IL6	NOS2	IL15	MX2	RTP4	CCL5	IL8	LAP	SLPI	TGM3	SAA3	LTF
Control	1	146	87	18531	490	1208	4086	938	39	529	2174	9332	20436	1664	67178
	2	40	61	17332	485	969	5369	910	32	578	4427	7513	19239	1411	65163
	3	164	97	24223	571	1351	2220	659	174	625	2106	10556	18539	2065	76821
Priming	1	205	117	25015	1074	1356	15388	4346	877	1475	20969	19764	44122	125698	252020
	2	247	135	18780	2023	1679	26259	6203	1226	1466	25170	25013	47420	139762	308480
	3	262	102	19653	1326	1526	18787	5149	902	1314	22615	19369	38228	118768	247731
Induction	1	16753	6918	127762	36615	8411	292093	28822	3240	36205	22191	14796	39490	326963	278773
	2	27561	9304	143679	62347	10168	412404	34509	1417	36205	27145	18385	34660	411939	442553
	3	18007	7412	120467	53129	9204	408286	36591	1937	37868	29275	17797	35342	383504	367726
Induction post priming	1	9841	5701	88559	14027	3933	70414	11813	1608	30842	42180	27790	76221	296961	520000
	2	11490	4947	78356	15752	4378	100892	15931	2079	29677	41131	30277	71053	283018	514841
	3	9296	4713	88058	14982	4145	101877	15016	1769	31040	42714	26794	76786	266950	538320
