# Supplementary material for: Lipopolysaccharide priming enhances expression of effectors of immune defence while decreasing expression of pro-inflammatory cytokines in mammary epithelia cells from cows
Source: BMC Genomics. 2012 Jan 12;13:17. doi: 10.1186/1471-2164-13-17 (PMC3315725; doi:10.1186/1471-2164-13-17)
Supplement: Additional file 5 — Table S5: Correlation of relative mRNA concentrations determined in microarray hybridizations or RT-qPCR from three biological pbMEC replica of the four challenge groups (C., P., I., and I.p.P.) of the short and long waiting experiments [file 1471-2164-13-17-S5.PDF]

Table S5: Correlation of relative mRNA concentrations determined in microarray hybridizations or qRT-PCR from three biological pbMEC replica of the four challenge groups (C., P., I., and I.p.P.) of the short and long waiting experiments.

Gene	SRC*
IL1B	0.90
TNF-α	0.86
IL6	0.93
NOS2	0.90
IL15	0.91
MX2	0.98
RTP4	0.96
CCL5	0.92
IL8	0.96
LAP	0.86
SLPI	0.77
TGM3	0.91
SAA3	0.95
LTF	0.95
**mean**	**0.91**

* Spearman's rank correlation coefficient
