# Supplementary material for: Lipopolysaccharide priming enhances expression of effectors of immune defence while decreasing expression of pro-inflammatory cytokines in mammary epithelia cells from cows
Source: BMC Genomics. 2012 Jan 12;13:17. doi: 10.1186/1471-2164-13-17 (PMC3315725; doi:10.1186/1471-2164-13-17)
Supplement: Additional file 6 — Table S6: Sequences of oligonucleotide primers used for real-time PCR quantification [file 1471-2164-13-17-S6.PDF]

Table S6. Sequences of oligonucleotide primers used for real-time PCR quantification.

Gene	GenBank no.	Primer sequence (5´–3´)	Amplificate size (bp)
IL1B	NM_174093.1	AACCGAGAAGTGGTGTTCTGC *	167
		TTGGGGTAGACTTTGGGGTCT *	
TNF	NM_173966.2	CTTCTGCCTGCTGCACTTCG	156
		GAGTTGATGTCGGCTACAACG	
IL6	NM_000600.3	GGAGGAAAAGGACGGATGCT	227
		GGTCAGTGTTTGTGGCTGGA	
NOS2	NM_001076799	ACAGGATGACCCCAAACGTC	188
		TCTGGTGAAGCGTGTCTTGG	
IL15	NM_174090	AACAGAGGCTGGCATTCATGT	240
		GTGGCATTTTTGGACTCGTG	
MX2	NM_173941	CACCTACCGCAACATTACGC	103
		GCTGATGCCAAGTCCATTCC	
RTP4	NM_001075961	GATGGACCCTGAAGATGGATG	107
		ACGGCATGAGGAACACTGG	
CCL5	NM_175827	TCCCCATATGCCTCGGAC	229
		TCGCACCCACTTCTTCTCTG	
IL8	NM_173925	CCTCTTGTTCAATATGACTTCCA	170
		GGCCCACTCTCAATAACTCTC	
LAP	NM_203435	AGGCTCCATCACCTGCTCCTT	182
		CCTGCAGCATTTTACTTGGGCT	
SLPI	NM_001098865	AATGTTGCCTCGACACTTGC	152
		TACGCACTGGTCGTCTGTCTC	
TGM3	NM_001101848	GCATGGATGTCACCGAAAAG	179
		TACACCAGCGACCTTGAACC	
SAA3	NM_181016.3	CTTTCCACGGGCATCATTTT	188
		CTTCGGGCAGCGTCATAGTT	
LTF	NM_180998.2	CTGTGGCTAAATTCTTCTCTGC	187
		TTAACAAAAGCCACGTCTCCAG	

*upper line: forward-, lower line, reverse-primer
